# New Approaches to the Computer Simulation of Amorphous Alloys: A Review

**DOI:** 10.3390/ma4040716

**Published:** 2011-04-13

**Authors:** Ariel A. Valladares, Juan A. Díaz-Celaya, Jonathan Galván-Colín, Luis M. Mejía-Mendoza, José A. Reyes-Retana, Renela M. Valladares, Alexander Valladares, Fernando Alvarez-Ramirez, Dongdong Qu, Jun Shen

**Affiliations:** 1 Instituto de Investigaciones en Materiales, Universidad Nacional Autónoma de México, Ciudad Universitaria, Apartado Postal 70-360, Mexico, D.F. 04510, Mexico; E-Mails: celaya@iim.unam.mx (J.A.D.-C.); terabyte_14@hotmail.com (J.G.-C.); amorpho@gmail.com (J.A.R.-R.); 2 Facultad de Ciencias, Universidad Nacional Autónoma de Mexico, Ciudad Universitaria, Apartado Postal 70-542, Mexico, D.F. 04510, Mexico; E-Mails: pedronavajas@gmail.com (L.M.M.-M.); renela6@yahoo.com (R.M.V.); avalladarm@unam.mx (A.V.); 3 Programa de Ingeniería Molecular, IMP, Eje Central Lázaro Cárdenas 152, Mexico, D.F. 07730, Mexico; E-Mail: falvarez@imp.mx; 4 School of Materials Science and Engineering, Harbin Institute of Technology, Harbin, Heilongjiang 150001, China; E-Mails: dqu2mse@yahoo.com.cn (D.Q.); junshen@hit.edu.cn (J.S.)

**Keywords:** amorphous alloys, computational simulations, bulk metallic glasses, electronic structure, vibrational densities of states

## Abstract

In this work we review our new methods to computer generate amorphous atomic topologies of several binary alloys: SiH, SiN, CN; binary systems based on group IV elements like SiC; the GeSe_2_ chalcogenide; aluminum-based systems: AlN and AlSi, and the CuZr amorphous alloy. We use an *ab initio* approach based on density functionals and computationally thermally-randomized periodically-continued cells with at least 108 atoms. The computational thermal process to generate the amorphous alloys is the *undermelt-quench* approach, or one of its variants, that consists in linearly heating the samples to just *below* their melting (or liquidus) temperatures, and then linearly cooling them afterwards. These processes are carried out from initial crystalline conditions using short and long time steps. We find that a step four-times the default time step is adequate for most of the simulations. Radial distribution functions (partial and total) are calculated and compared whenever possible with experimental results, and the agreement is very good. For some materials we report studies of the effect of the topological disorder on their electronic and vibrational densities of states and on their optical properties.

## 1. Preamble: Atomic Topology *versus* Properties

It is an obvious fact that the atomic constituents of matter, their interactions and their spatial arrangements determine the properties of the material. It is desirable then to look at the structure of a substance in order to have a close idea of the properties that one should expect when dealing with it. This explains the development of experimental techniques that early on gave a picture of the distribution of atoms long before there were theoretical advances as a necessary counterpart. This is especially true when dealing with complex materials, like the amorphous and the porous, where analytical developments were at best applicable to only a very restricted class of substances. Computational simulations had to come of age for them to be really helpful and useful, *ab initio* techniques had to be further developed to provide results that would not be parameter dependent or potential dependent. But not everything is that simple. *Ab initio* methods are very computer demanding which restricts the size of the samples that can be studied with these methods to at most only a few hundred atoms. This implies that nanoscopic structures can be dealt with reasonable confidence, but mesoscopic properties will have to wait until the first principles methods are further optimized to be able to handle larger number of constituents and defects. There are also a notable amount of ‘approximations’ that depend either on the particular method used, or the type of wave functions, or whether full core or pseudopotentials are incorporated in the calculations, since these determine the quality of the approximation.

Another factor to be considered is the fact that the calculations have to be carried out on a large enough number of atomic constituents to guarantee results that represent the extended structure, otherwise the limited sample may not be representative of the bulk since boundary or surface effects may significantly alter the outcome of a calculation. To palliate these deficiencies the supercell approach has been developed which consists in constructing large atomic cells, with amorphicity or porosity, subject to periodic boundary conditions that extend them throughout space giving a ‘false’ sense of a material in the bulk. Evidently the size of the supercell that can be handled with *ab initio* techniques is a limiting factor when dealing with extended mesoscopic defects. Strictly speaking an amorphous material would have to have a supercell that contains some 10^23^ atoms since its structure is not repetitive and since there are no simplifying Bloch-like theorems-like in the crystalline state—that would allow handling smaller but nevertheless representative atomic arrangements; a description of such a large structure would be impossible by any means. So what is there to do? It turns out that supercells that contain a number of atoms much smaller than 10^23^ can give us realistic information of the short and middle range ordering that may exist in a material, since the lengths involved would be of the order of tens of angstroms, which is accessible in a first principles computational simulation.

Thus, in non-crystalline bulk materials and with present day techniques the *ab initio* approximations can be used to describe:

➢ Small nanoscopic defects
➢ Short and middle range ordering

If this is combined with the presence of periodically extended large supercells we can hope to create an approximate representation of amorphous materials since long range ordering is absent in these disordered atomic structures. Periodic supercells are frequently used in the field of computer simulations of complex (amorphous, porous, liquids, *etc*.) materials, acknowledging the impossibility to deal with samples large enough to be fully representative. This leads to the appearance of a spurious periodicity in the simulations that is extrinsic to the problem.

But how do we go about generating an amorphous supercell? Or a porous supercell for that matter? Regarding amorphicity, we have previously reported successful calculations using a new approach that we have denominated the *undermelt-quench* approach [1]. Regarding porosity we developed a simple process that we named the *expanding lattice* approach to generate amorphous nanoporous structures [2]. Both techniques are innovative and useful to describe a certain class of materials. In this review work we concentrate on the approach used in Reference 1, and report some of our results, some new and some already published, for amorphous semiconducting and metallic alloys.

## 2. Antecedents [1]

The generation of amorphous topologies has a long history of approaches using a variety of force fields and different dynamical approximation methods. Since crystalline semiconductors provoked a revolution in electronics it is understandable that amorphous semiconductors were extensively produced and studied experimentally. This in turn provoked the development of theoretical models that contributed to the understanding of the atomic structure of these disordered materials. Amorphous metallic systems are on the other hand very difficult to produce in the laboratory since they tend to crystallize rapidly because the amorphous phases can be very unstable. In some materials, high cooling rates of ~10^6^ K/s are needed to bypass crystallization and this restricts the thickness of the samples obtained to a few micrometers [3]. The first attempts at producing metallic glasses were done by metal deposition on cold substrates which invariably led to thin film samples. Metallic glasses obtained by rapidly quenching melts were reported in 1960 by Klement *et al*. who quickly cooled an Au–Si alloy from about 1,300 °C to room temperature leading to samples in the micrometer regime [3]. *Ab initio* modeling of metallic systems has been widely used to study local structures in pure and alloyed liquid metals [4], but its application to the generation of amorphous atomic topologies of solid metallic alloys has been very limited due perhaps to the small number of atoms that can be dealt with, despite their potential and necessary applicability to Bulk Metallic Glasses (BMG). Recent work reports the use of the Honeycutt-Anderson method to analyze amorphous alloys generated via computer simulations [5].

For amorphous systems the attempts to generate reasonable atomic topologies can be classified via two extremes: (i) calculations based on *ad hoc* classical, parameter-dependent potentials, constructed for the specific purpose of generating amorphous samples of certain materials; (ii) quantum methods, parameterized and *ab initio*, that can deal from the outset with the thermalization processes used to generate the amorphous structures; with the interactions among electrical charges that lead to their structures and lead to their electronic and related properties, to understand their physical and chemical behavior. Much simulational work has been carried out both on pure elements and on alloyed systems [1]. However, for the purposes of the present work we shall ignore some of the results we have obtained for pure elements and will concentrate on the description of amorphous semiconducting and metallic alloys.

For solid amorphous metallic systems the generation of disordered structures using first principles techniques is to our knowledge non-existent. There is some published work on the properties of liquid metallic systems: pure elements, alloys, and semiconductors, like Si and Ge, which are metallic in nature when in the liquid state. In what follows we report some of our unpublished results on the generation of amorphous metallic alloys in the solid state.

Since 1985 Car-Parrinello molecular dynamics [6] and quenching from the melt of periodically-continued supercells have been extensively used to *ab initio* generate amorphous structures of covalent semiconductors. Without doubt the pioneering work of Car and Parrinello has been a landmark in the development of the field, and has permeated most efforts up to the present. This technique of quenching from the melt is frequently used and in the literature is commonly known as the *melt-and-quench* approach; it invariably generates a large number of bond defects, floating or dangling, in amorphous semiconductors when their liquid phases are metallic with larger coordination numbers. There is another common approach to the generation of amorphous substances in which ‘perfect’ random networks are constructed by hand, by switching bonds and adjusting plane and dihedral angles, where no bond defects are incorporated. The two procedures are opposite and only partially represent real amorphous materials. So it was necessary to search for a different thermal procedure that avoids the melting history of the first process and the ‘perfect’ construction of the second, hence the *undermelt-quench* approach that we have developed [1].

Car, Parrinello and collaborators applied their first-principles plane-wave molecular dynamics method (Car-Parrinello Molecular Dynamics, or CPMD) to C, Si, and Ge. Their simulations were done starting from the corresponding liquid phases and, after cooling them, radial distribution functions (RDFs) were calculated for the range 0 < *r* < *l*/2, where *l* is the length of the supercell edge used and generally includes the first two radial peaks. Even though the RDFs obtained reproduce reasonably well the first two peaks of the experimental results, the overall agreement with experiment varies from material to material. Furthermore, the procedure of quenching from the melt produces a large number of overcoordinated atoms since some of the liquid phases of these semiconductors are metallic; e.g., liquid silicon and liquid germanium have average coordination numbers between 6 and 7, and the quenching from the melt preserves some of this overcoordination. This excess of bond defects makes the electronic and/or optical gaps difficult to observe.

Chronologically, the first application of CPMD was to amorphous Si, *a-*Si [6] and then to liquid silicon [7,8] and most of the existing calculations stem from this original work [7,8,9,10,11,12]. Car and Parrinello performed this first *ab initio* molecular dynamics (MD) study on an fcc periodic supercell with 54 atoms of silicon using their plane wave MD method. In their approach a non-local pseudopotential was used together with the parameterized local density approximation (LDA) form of Perdew and Zunger for the exchange-correlation effects [7]. They obtained good agreement up to the second radial peak, with the experimental RDF of *a*-Ge rescaled to simulate *a*-Si, and argued that because of the size of their supercell, distances larger than 6 Å could not be studied. They pointed out that comparisons of simulated and experimentally determined atomic structures should be carried out with care in view of the large number of defects generated in the simulation. A typical simulation for *a-*Si was started above the melting point at about 2,200 K, and the liquid was allowed to equilibrate for ≈ 0.7 ps before it was quenched down to ≈300 K at a cooling rate of ≈2 × 10^15^ K/s. During the initial quenching the volume of the cell was gradually changed to 1080 Å^3^, the crystalline value.

Since then several works have appeared that generate disordered structures using CPMD and the *melt-and-quench* procedure. We now present a brief résumé of the pertinent works for our purposes, introducing the nomenclature where generally *a*-AB refers to the family of amorphous alloys that have varying contents of the elements A and B, whereas *a*-A*_x_*B_1*−x*_ or *a*-A*_y_*B*_z_* refer to a specific alloy. Sometimes we shall use the nomenclature *a*-AB*_x_* just to be consistent with the established identification procedure of having an amorphous alloy that has 1 part of element A for *x* parts of element B.

Let us begin with amorphous hydrogenated silicon. Simulating *a-*SiH is a difficult task since there seems to be a strong dependence of its atomic topology on deposition conditions; also, the chemical reactivity of hydrogen is another factor that has to be addressed. Finally, the high mobility of H compared to the mobility of Si, and the role of its zero point energy have to be taken into account, indicating the necessity of a quantum mechanical *ab initio* approach to this material. The corresponding CPMD work is due to Buda *et al.* where the plane-wave Car-Parrinello method was applied to an amorphous hydrogenated cubic cell of 64 silicons and 8 hydrogens (11% concentration) [13]. These authors started out with a liquid material containing both silicon and hydrogen atoms which was rapidly quenched, maintaining a density equal to the value of the crystalline material. They report only partial distribution functions and the H-H RDF obtained in these simulations compares poorly to the existing neutron scattering experiments. We believe that the poor agreement is mainly due to the fact that the simulational supercells are melted before being solidified. Fedders and Drabold [14] and Tuttle and Adams [15] have also studied *a-*SiH from first principles. Fedders and Drabold do not report any RDF, total or partial, whereas Tuttle and Adams report only the Si–Si and the Si-H RDFs of a cell of 242 atoms with 11% hydrogen. Tuttle and Adams generated their structures from a liquid at ≈1,800 K, which was then quenched to produce an amorphous sample at 300 K. They assumed that the mass of each hydrogen atom *equals* the mass of each silicon atom leading to an unrealistic representation of the diffusion of hydrogen in the sample. This unrealistic assumption implies an unrealistic H-H RDF so perhaps that is why no H-H RDF is reported in their work. Furthermore, the high mobility of the hydrogen atoms relative to the mobility of the heavier silicons makes the handling of an adequate time step in the simulation more difficult.

There were no CPMD-based calculations for *a-*SiN before the publication of the results of our group in 2002, results that shall be presented in Section 4 and Section 5. As far as we know *a-*SiN alloys have not been the subject of any other type of quantum molecular dynamics simulations up to the present, and therefore our work was the first, and so far it is the only *ab initio* study of this material.

For *a*-CN, there are some *ab initio* studies using the CPMD approach [16,17]. McKenzie and coworkers use random networks that were generated by the *melt-and-quench* method on a crystalline 64-atom supercell. They studied the electronic density of states to investigate the probable doping mechanism of carbon by nitrogen. Only three different densities were considered, each for two concentrations, influenced by the experimental work of Walters *et al.* [18]. The two concentrations were C_62_N_2_ and C_56_N_8_, and the three densities were 2.45, 2.95 and 3.20 g/cm^3^. An additional simulation was carried out for a density of 2.7 g/cm^3^ and a concentration of C_60_N_4_. They claimed that, contrary to the experimentally found substitutional doping, their results did not indicate that for low concentrations nitrogen behaves as a dopant in amorphous carbon.

The first CPMD simulation concerning the structure of amorphous silicon carbide, *a*-Si_0.5_C_0.5_, was reported in 1992 by Finocchi *et al.* [19]. They performed CPMD on two different samples, one with 27 C and 27 Si atoms randomly distributed throughout the diamond crystalline positions, and the other with 32 C and 32 Si atoms randomly distributed throughout the rock-salt crystalline structure positions. Both samples have a density of 3.1 g/cm^3^. The authors used a *melt-and-quench* procedure where the samples were heated up to a temperature of approximately 4,000 K, then they were equilibrated during a 1 ps interval and cooled down to approximately 500 K. The authors affirmed that the two samples had very similar structural properties, thus they concluded that the simulations were not dependent on the initial structures. The electronic properties of these samples were calculated at the Γ point, despite the small number of atoms and the small size of the supercells. In this work the authors reported the total and partial RDFs, and the electronic density of states (eDOS). The authors observed that the RDFs had a peak around 1.5 Å due to C–C bonds and 1.9 Å due to Si-C bonds, so the first coordination shell was formed by two different bond types, also 40–45% of the bonds present in the sample were C–C homonuclear bonds. Two conclusions were drawn in the analysis of the electronic structure of the amorphous samples: the first was that the material is a semiconductor; the second was that the ionicity gap observed in crystalline silicon carbide, *c*-SiC, located at about −11 eV with respect to the Fermi level disappears.

The second CPMD work was also done by Finocchi *et al.* and had the objective of studying the local atomic environment of *a*-Si_0.5_C_0.5_ [20]. Here the authors generated the amorphous structure of SiC using a technique very similar to the previous work [19]. In this paper they could not establish a sample structure either chemically ordered or completely random. The general remark was that a detailed analysis of each atomic species is of vital importance in order to understand its physical properties.

In order to explore the local atomic environment of *a*-(SiC)H, Finocchi and Galli in 1994 worked out a CPMD amorphization of a simple cubic supercell of 3.18 g/cm^3^ which was made up of 32 C, 32 Si and 12 H atoms [21]. The *a*-(SiC)H sample was produced by a rapid quenching from the melt, at ~4,000 K, to 500 K, using the same procedure as in Reference 19. The authors reported the total and partial distribution functions, and the coordination numbers, but since we have not studied this system we shall not dwell on it.

There are a few CPMD studies on amorphous GeSe_2_. Recently, Massobrio and Pasquarello generated amorphous networks by cooling a 120-atom liquid supercell [22,23]. They used 40 Ge and 80 Se and a periodic cubic supercell with an edge length of 15.16 Å and a density of 4.38 g/cm^3^. There are *ab initio* works based on the Harris density-functional method developed by Sankey and coworkers [24]. In the first work, Cappelletti *et al.* studied the vibrations in amorphous GeSe_2_ using a diamond-like supercell of 63 atoms, 20 Ge and 43 Se [25]. They applied the *melt-and-quench* process to their model and did some removing and adding of atoms to end up with exact stoichiometry: 21 Ge and 42 Se, and a resulting density of 4.20 g/cm^3^. They compared their vibrational density of states (vDOS) results with the experimental one. In the second work on *a*-GeSe_2_, Cobb *et al.* report a 216-atom supercell [26]. In this *ab initio* study they reported topological, vibrational and electronic properties that will be compared with our results later on.

For completeness, and to the best of our knowledge, there is no CPMD work reported on *a*-InSe, but tight-binding calculations were performed by Kohary and collaborators [27]. They amorphized In-Se with different densities and numbers of atoms per supercell, depending on the basis set: 64 and 124 atoms for DNP and SN basis sets. These cells were made amorphous by the *melt-and-quench* procedure. The positions of the first peak were in the range of 2.60 and 2.76 Å. They concluded that there were no Se-Se homopolar bonds for *a*-In_0.5_Se_0.5_.

No CPMD works on aluminum-based alloys were found, except for a calculation of Alemany *et al.* where the Kohn-Sham *ab initio* molecular dynamics is applied to study liquid aluminum near the melting point [28]. Our own work, which shall be presented at a later stage, is based on the Lin-Harris MD, LHMD, and is not self consistent as the CPMD.

Finally, theoretical studies via simulational modeling exist for the glassy system CuZr, *g*-CuZr, but not for the *amorphous* CuZr, *a*-CuZr. Wang *et al*. performed some *ab initio* molecular dynamics (AIMD) and reverse Monte Carlo studies (RMC) on a sample with the same concentration they had used for their X-ray diffraction (XRD) analysis [29]. By comparing the AIMD with the XRD results, and RMC with extended X-ray absorption fine structure (EXAFS), they obtained the 3D structure of the samples from which they established the short range ordering. Likewise, Mattern *et al*. carried out an RMC study to resolve the partial radial distribution functions (pRDFs) and consequently the coordination number [30]. In addition to this, Jakse and Pasturel have reported an AIMD study for the Cu_64_Zr_36_ alloy [31,32]. They obtained a coordination number closer to the one found by Mattern and co-workers, *i.e.*, 13.1. It is noteworthy that these computational works have in common the use of plane waves as basis sets and a thermal procedure which leads to obtaining the metallic glass cooling from the melt. Also, neither the AIMD studies (except for the work of Jakse and Pasturel) nor the values reported experimentally establish clearly the method they used to compute the coordination numbers.

Another work on the *g*-CuZr system, worth mentioning although it is not *ab initio*, is the one done by Sun and coworkers where the Finnis-Sinclair potential was used [33]. A complete study of the temperature effects on the structural evolutions and diffusivity of this alloy was conducted. In particular, the pair distribution functions and common-neighbor analysis were used to investigate the structural variations. Also, the mean square displacement and the self part of the van Hove function were calculated to evaluate the relaxation and transport properties. Finally, the critical temperature *T_c_*, a predicted glass transition temperature for Cu_60_Zr_40_ glass former, is calculated to be 1,008 K. An interesting challenge is to see what the results would be when using an *ab initio* approach to this problem.

It should be clear that, before our incursion in the field, the *ab initio* amorphization of crystalline supercells was essentially based on the *melt-and-quench* procedure that had been in use in the literature since both classical and quantum computer simulations began to appear. It should also be clear, as mentioned before, that because the group IV semiconductors are metallic in the liquid state, melting them leads to the appearance of extra bonds in the atomic coordination of the liquid state so that when the supercells are quenched, some of this overcoordination is carried over into the disordered solid phase. The result is that the samples so generated are not fully representative of the experimental samples obtained by techniques other than melting and quenching.

Our contribution to the field of amorphous semiconductors is having devised a new method: the *undermelt-quench* approach, which does not melt the crystalline supercells but heats them to just *below* the melting temperature, avoiding the liquid state and consequently the overcoordination that occurs in this phase compared to the coordination of the crystalline or amorphous solid phases. Clearly, the code used was also a determining factor (Fast Structure Simulated Annealing by Molecular Simulations, Inc.) since it was devised for finding in a rapid manner the minimum energy structures of atomic aggregates after having disordered them *stochastically*. Because of this, our results are more representative of the experimental ones and agree better with them. In Section 3 we present this method, and its variants. Using our approach, in Section 4 we review results that we obtained, and already published, for the following amorphous systems: SiH, SiN, CN, new results are reported for silicon carbide, for the chalcogenide binary alloy *a*-GeSe_2_, for aluminum-based alloys, *a*-AlN, *a*-AlSi, and for *a*-CuZr. In Section 5 we present some physical properties calculated for some of these amorphous systems. In particular, we report: the electronic density of states of the hydrogenated amorphous silicon samples; the optical gaps of the amorphous silicon nitrogen alloys using an approach *a la* Tauc devised by us and the vDOS using a large, 216-atom amorphous silicon sample that lead to results in remarkable agreement with experiment. Section 6 contains general conclusions.

It remains an issue as to how generally and how accurately an *ab initio* method that uses a relatively small supercell can describe the properties of amorphous materials both semiconducting and metallic. The present work addresses this issue and presents new alternative methods to computer generate disordered atomic topologies of alloys that agree very well with experiment, when available, as long as the defects present are not in the mesoscopic regime and as long as the properties studied are mainly dependent on the short and middle range order of the material.

## 3. The *Undermelt-Quench* Approach [1], Its Variants, the Method and the Bonding Criterion

The new amorphizing thermal procedure described here, was developed by one of the authors (AAV) while spending a sabbatical year (1997) at Molecular Simulations Inc, MSI, (now known as Accelrys, Inc.), in San Diego, CA, USA, since their code Fast Structure Simulated Annealing (Fast for short) [34] seemed appropriate to generate bulk amorphous structures. The code was developed by John Harris and collaborators to fast find the structure of atom aggregates, but the periodic boundary conditions incorporated, and the initial randomness in the atomic velocity distribution gave it a broader applicability [35].

The *ab initio* methods attempt to answer questions from first principles and are generally applicable without adjustment of parameters. Since these methods are very demanding on computer resources they are presently limited to handling a relatively small number of atoms; *i.e.*, to relatively small supercells. There is also a notable amount of ‘approximations’ that depend on the particular method used, or on the type of wave functions, or whether full core or pseudopotentials are incorporated in the calculations, or the ‘parameters’ that appear when using approximations for the exchange-correlation interaction, to mention those that occur most commonly and that determine the quality of the approximation. Within the first principles methods one has the option of using a recursive self-consistent approach [36,37] like the one used in CPMD, or using a linear combination of atomic orbitals, LCAO, non self-consistent approach employing a functional like the one developed by Harris [38] and implemented in the LHMD. In general the self-consistent method is more computer-demanding than the non self-consistent one.

Fast, the code that we used at the beginning is, in short, a density functional code based on the Harris functional, which generates energies and forces faster than traditional Kohn-Sham functional methods, since the code is not self-consistent. However, it is not always possible to use the Harris functional since, until recently, it was unable to handle partially filled d-band materials. New developments that shall be mentioned later on seem to remedy this situation. In all our calculations we have used the LDA with the parameterization of Vosko, Wilk and Nusair (VWN) [39]. Some of our calculations are performed on an all electron basis, some use pseudopotentials, particularly when heavy atoms are considered. The valence orbitals are described either viaminimal or standard basis sets of ‘finite-range’ atomic orbitals with a cutoff radius chosen as a compromise between computational cost and accuracy, and in general are different for the various materials. Fast, and later developments of the Code, can handle three types of basis sets: (i) minimal, consisting of the atomic orbitals occupied in the neutral atom; *sp*-valence type; (ii) standard, broadly equivalent to a Double Numeric basis set, DN and (iii) enhanced, broadly equivalent to a Double Numeric set together with Polarization functions, DNP. The computation scales with a high power of the cutoff radius, because the time-limiting factor is the number of three-center integrals that have to be carried out [40,41]. The linear combination of atomic orbitals utilized makes the minimum energy atomic structures generated very close to experiment. The interatomic distances fall within 1% of the experimental values for a large variety of small molecules [34,35]. Fast uses optimization techniques through a force generator to allow simulated annealing/molecular-dynamics studies with quantum force calculations. The forces are calculated using rigorous formal derivatives of the expression for the energy in the Harris functional, as discussed by Lin and Harris [42]. Three-center integrals were performed using the weight-function method of Delley [40,41] with correction for the dependence of the mesh on the nuclear coordinates. This is the essence of Fast, the code developed by Harris *et al.* for MSI.

Fast initially disrupts the atomic aggregates stochastically, then heats them using molecular dynamics to foster the rearrangement of their atomic constituents and finally cools them to what would be the structure of minimum energy, at least locally. Rather than using this code to find the minimum-energy atomic structure of a cell, we use it to generate random structures from an originally crystalline supercell with periodic boundary conditions.

**Figure 1:**
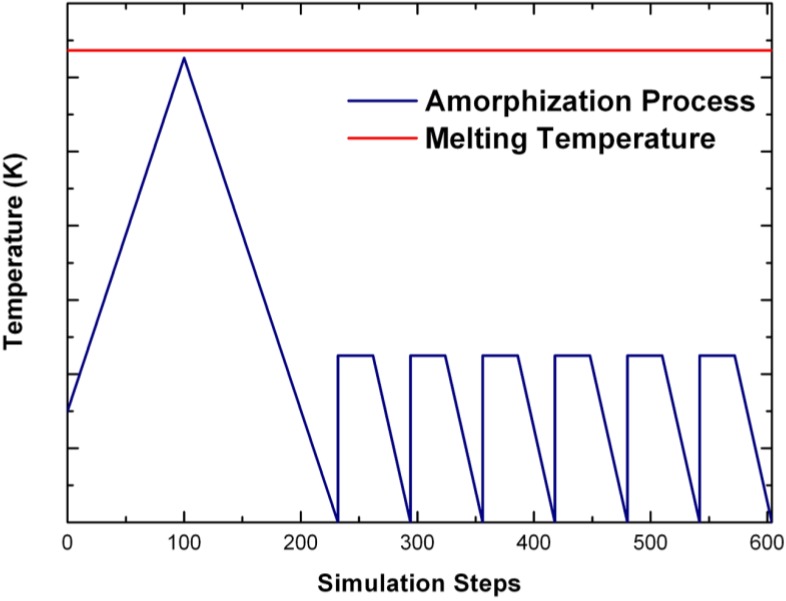
Schematic representation of the original *undermelt-quench* approach. The first process (triangular) is the randomizing part; the rest are the stress-relieving cycles. At the end a geometry optimization is carried out. The temperatures considered are specific to each material. For variants of this process see text.

The difference between our approach and previous techniques resides in the heating procedure and in the code used. It is clear from previous work that quenching from a melt, or from partially melted samples, generates structures that only partially resemble the local arrangement of atoms in the amorphous material; therefore, we took a different route. A corresponding crystalline supercell with the chosen number of atoms and the same density as the amorphous phase is subjected to the following process. The supercell is heated from 300 K to *just below* the corresponding melting temperature (the liquidus temperature in binary systems) in 100 steps, with a time step three to four times the default value. It is then immediately cooled down to 0 K in the necessary number of steps required by the cooling rate which is the same in magnitude as the heating rate. This is the proper amorphizing stage. Physical masses of the atoms are used throughout and this allows realistic atomic diffusive processes to occur in the system, and lets them move within each periodic supercell. Once this first stage is complete, several variants have evolved. In the original process each supercell is subjected to six annealing cycles at a temperature dictated by experiment, with intermediate quenching processes, in order to release the stresses generated. Finally, a geometry optimization is carried out. Other variants have been developed to describe specific systems. If the atoms are heavy, like in the chalcogenides or the BMG-like systems, the stress releasing cycles are reduced to one, or none, to minimize computational times but the structure optimization process stays. Finally, a plateau is sometimes introduced at constant temperature either below the melting point or above the melting point, depending on the material to be disordered and the process desired, *i.e.*, this constant temperature is the value at which the system is maintained long enough to foster the amorphizing or the liquefying procedure. Then the system is cooled rapidly, usually in one step, or maintained at this temperature. The original, amorphizing process will generically be referred to as the *undermelt-quench* approach with its variants. The original process is shown schematically in Figure 1.

Since Fast initially disrupts the atomic aggregates randomly, the probability of returning to a crystalline structure after the initial heating and cooling (amorphizing) cycle is nil for semiconductors. One might think that our approach may be dependent on the initial crystalline structures used, but we have demonstrated that the RDFs generated starting with diamond-like low density carbon structures are practically indistinguishable from those obtained from initial hexagonal or rhombohedral structures [43,44].

In the present work the amorphizing procedure was performed self-consistently for CuZr, since the Harris functional lacks the tools to deal with partially filled d-band metals. However, recent developments indicate the possibility of generalizing the Harris functional to deal with these metals [45]. Sometimes energy calculations were carried out using both Fast and the full Kohn-Sham DFT approach [36,37] implemented in the *ab initio* DMol3 commercial code [40,41,46] to obtain the eDOS curves of the final amorphous atomic structures.

It should be clear from the outset that our computational processes do not pretend to mimic the production of such materials, but only to generate random networks, using *ab initio* techniques, that lead to RDFs that agree with experiment; this agreement allows us to study their topological, electronic, optical and vibrational properties.

Also, since we use at least 108 atoms almost everywhere, we have Fourier-smoothed the RDFs to have adequate curves to allow comparison with experiment. The number of atoms used is low and this leads to statistical fluctuations that are not representative of the bulk.

Finally, we would like to mention the criterion that we have used to determine the ‘extension of a bond’. Bonds, being electrical in nature, have in principle an infinite range, but in practice they have a finite range, so how is one to define when two atoms are bonded? This is a difficult problem in amorphous materials and some authors have carried out extensive searches for possible molecular (cluster) structures of a given element, silicon for example, to infer a probable bond length [47]. Others opted for the use of localized wave functions, like the Wannier-type, to get an estimate of the bond lengths [16]. One can also look at the charge distribution between atoms and set a limit below which the bonding is declared nonexistent. We decided to use throughout this work a geometric approach to the bonding problem. We believe that the structure of radial distribution functions is a manifest way to determine the *maximum* bond length, especially when there is a clear zero minimum between the first and the second peaks which is the case for most elemental, monatomic, amorphous semiconductors. When amorphous alloys are considered, a way to determine the bond lengths among the diverse species is by looking at the minimum between the first and second peaks of the corresponding pRDFs; the maximum bond lengths are then set equal to the position of these minima. Using this approach we determine the extension of the bond, and by integrating the area under the corresponding peak of the adequate RDF we can also calculate the number of neighbors, although with this procedure it is difficult to determine the multiplicity of the bonds (single, double or triple) in *a-*semiconductors.

## 4. Amorphous Alloys: Their Atomic Topologies

In what follows we shall discuss the amorphization of several alloys, both semiconducting and metallic. We also present the atomic topology of some chalcogenide alloys. Some of these amorphous alloys have been studied before by means of the more conventional methods mentioned above and it is desirable to compare our results, obtained with Fast or with DMol3 and using the *undermelt-quench* approaches, with previous ones. Both, total and partial RDFs are presented to illustrate the relative atomic organization. For some systems coordination numbers and neighbor analysis are reported.

### 4.1. Amorphous Hydrogenated Silicon [1,48]

The experimental hydrogenation of amorphous silicon played a decisive role in the development of amorphous semiconductors as useful materials for the electronic industry. However, the computer simulation of this ‘alloy’ has been difficult and our success is doubtless due to the procedure that we followed and is described next.

#### 4.1.1. Preamble

Amorphous hydrogenated silicon, *a-*SiH, was a turning point in the technological applications of amorphous semiconductors. The passivation of dangling bonds of amorphous silicon with hydrogen opened the door to the doping of amorphous semiconductors. This in turn allowed the control of their electrical properties with a direct incidence in their use, analogous to the way in which the *n-* and *p-*doping of crystalline semiconducting materials revolutionized their use in technology.

The two amorphous networks generated for pure silicon [1,48], one with the default time step of 2.44 fs, and the other with approximately four times the default time step, 10 fs, were used as starting points for constructing cells of *a-*SiH. Although the short time step simulation gave an amorphous silicon structure in poor agreement with experiment, it was not necessarily a bad starting point for preparing a hydrogenated sample. Hydrogen atoms tend to decorate dangling bonds, and the shorter time simulation generated more dangling defects. Thus, both configurations of atoms were used in attempting to generate an acceptable hydrogenated structure. One simulation was performed with each *a*-Si sample, retaining consistency with the time interval between steps. Starting from the 2.44 fs sample, we used the physical hydrogen default time step of 0.46 fs for the subsequent evolution. We refer to the resulting structure as the 2.44/0.46 cell. With the second simulation, performed using the 10 fs *a*-Si as starting point, we used a 2 fs time step for subsequent hydrogen evolution; this structure is referred to as the 10/2 cell.

**Table 1:** Fractional initial positions of the 12 hydrogens placed symmetrically within the silicon supercells.

Relative starting positions of the hydrogen atoms in the amorphous silicon cells	
H1	(1/4, 1/4, 1/4)
H2	(3/4, 1/4, 1/4)
H3	(3/4, 3/4, 1/4)
H4	(1/4, 3/4, 1/4)
H5	(1/2, 1/4, 1/2)
H6	(3/4, 1/2, 1/2)
H7	(1/2, 3/4, 1/2)
H8	(1/4, 1/2, 1/2)
H9	(1/4, 1/4, 3/4)
H10	(3/4, 1/4, 3/4)
H11	(3/4, 3/4, 3/4)
H12	(1/4, 3/4, 3/4)

For hydrogenated amorphous silicon two different procedures were implemented. First the amorphous pure silicon cell generated with a time step of *t* = 2.44 fs was used and then it was expanded to a volume of (11.0620 Å)^3^ to reproduce the experimental density, 2.2 g/cm^3^ of the hydrogenated structure with 12 hydrogens. Second, a previously expanded crystalline cell of 64 silicon atoms with the same volume of (11.0620 Å)^3^ was amorphized using a 10 fs time step. We then placed the 12 hydrogens evenly distributed throughout the amorphous cells. The starting locations of the hydrogen atoms in the cell are given in Table 1. The hydrogenated samples were then subjected to annealing cycles using a time step of *t* = 0.46 fs for the first cell (the 2.44/0.46 cell), and *t* = 2 fs for the second cell (the 10/2 cell). The annealing cycles consisted of two cycles of 50 steps at 300 K for the large time step sample and one cycle of 200 steps for the small time step sample, with in between quenches down to 0 K, to allow the hydrogens to diffuse and move within the cells. This gave a concentration of hydrogen of practically 16%, adequate to compare with existing experimental results.

**Figure 2:**
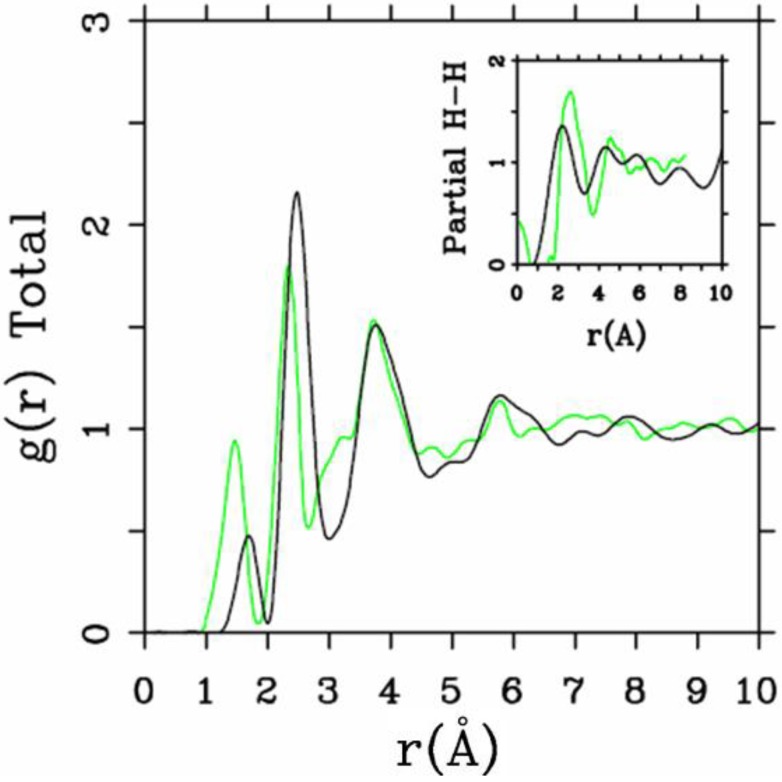
Direct comparison of the simulated total radial distribution function (RDF) and H-H pRDF (Inset) for a-SiH (black lines) to the experimental results (green lines) for the 2.44/0.46 fs cell. These simulations give a better overall description of the observed H–H pRDF.

#### 4.1.2. Results and Analysis

**Figure 3:**
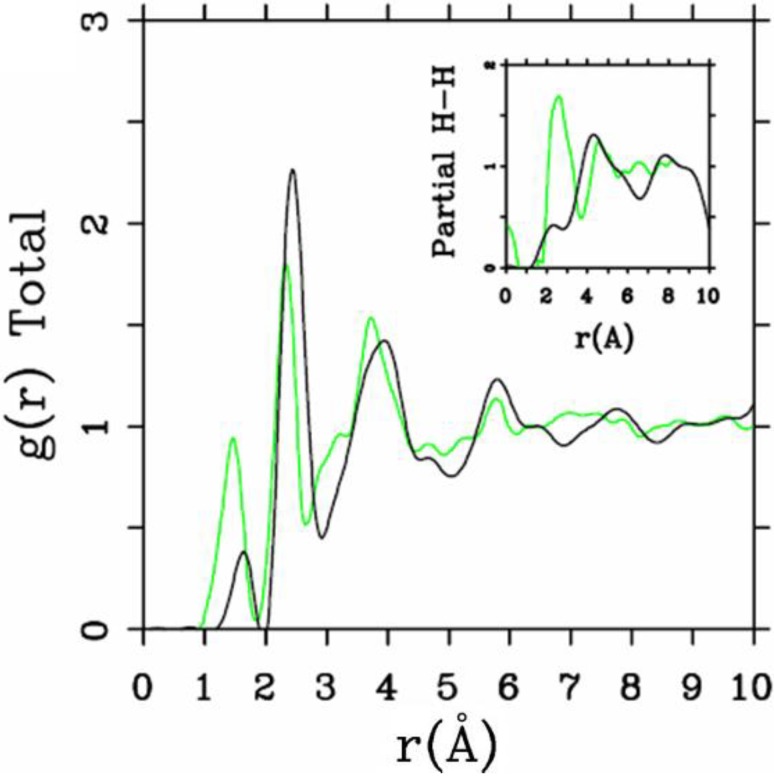
Direct comparison of the simulated total RDF and H–H pRDF (Inset) for *a*-SiH (black lines) to the experimental results (green lines) for the 10/2 fs cell. The peak due to molecular hydrogen is not shown.

One important difference between the two simulations was the formation of ‘molecular hydrogen’ in the 10/2 case. The reason for this is the higher number of coordinated Si atoms in this sample, which leaves insufficient opportunities for the H-atoms to bond to silicons. In the 2.44/0.46 case, the defect/dangling bond density was sufficiently high for all hydrogens to find good bonding locations in the *a-*Si environment. Direct comparisons with experiment for the two cases are shown in Figure 2 and Figure 3, where in the latter case the peak due to di-hydrogen (molecular hydrogen) was not included.

While both simulations represent the main features of the measured total RDF reasonably well, the H–H pRDF is much better represented in the 2.44/0.46 simulation than in the 10/2 simulation. The experimental data of Bellissent *et al.* [49] are better reproduced by the originally highly defective sample of *a*-Si with added hydrogen. The experimental features observed for *r* ≈ 0 are spurious [49].

**Figure 4:**
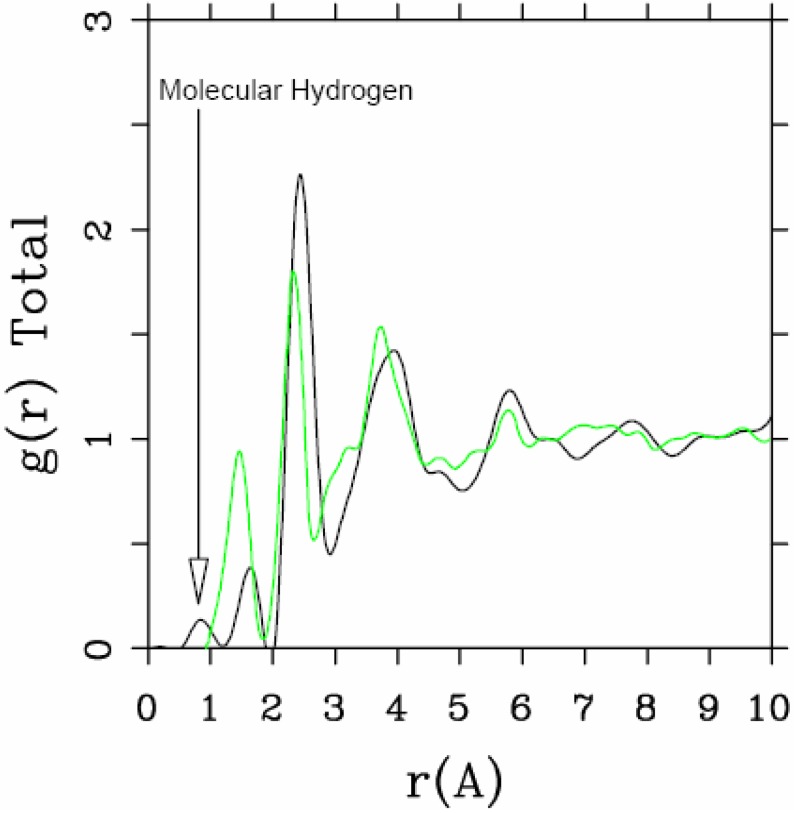
Total RDF for the 10/2 fs cell of *a*-SiH (black line) that shows the presence of molecular hydrogen, compared to the experimental results (green line).

The presence of di-hydrogen in the 10/2 simulation is illustrated in the total RDF in Figure 4. The molecular hydrogen peak appears at an interatomic distance of 0.875 Å, very close to the molecular radius of 0.86 Å found for molecular hydrogen in crystalline silicon [50].

**Figure 5:**
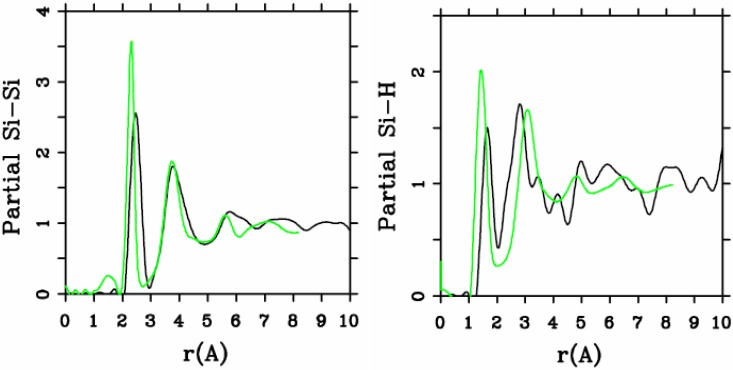
Partial Si–Si and Si–H RDFs for the hydrogenated 2.44/0.46 cell. The black lines are our simulation and the green lines are the experimental results.

**Figure 6:**
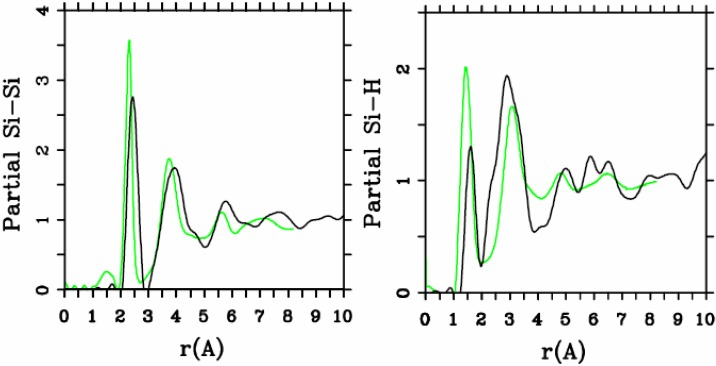
Partial Si–Si and Si–H RDFs for the hydrogenated 10/2 cell. The black lines are our simulation and the green lines are the experimental results.

Figure 5 and Figure 6 represent pRDFs for Si–Si and Si–H for the hydrogenated 2.44/0.46 cell and for the 10/2 cell. The overall agreement between the experimental and simulated Si–Si and Si–H pRDFs is quite reasonable as the direct comparison indicates. In this case, there is no strong distinction between the two simulations.

**Figure 7:**
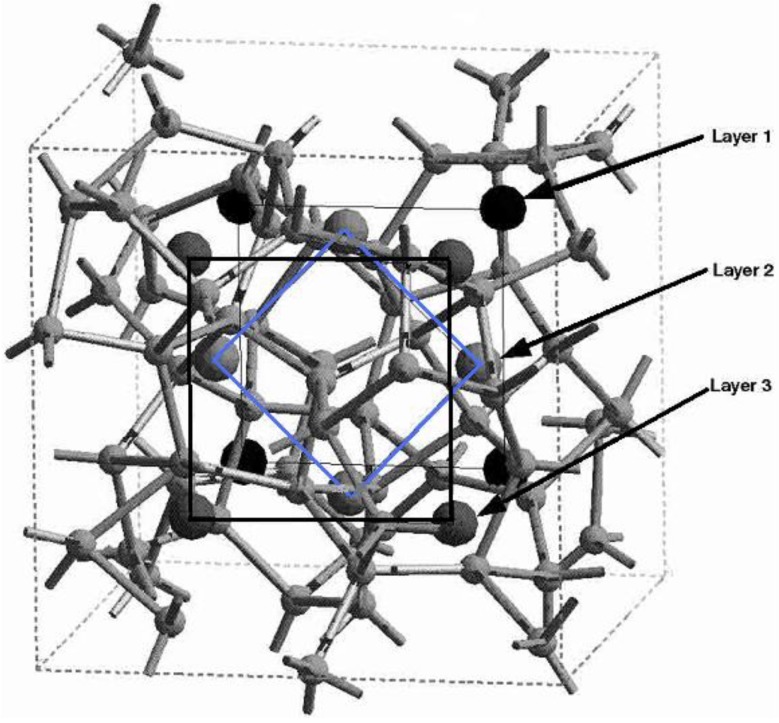
The initial location of the 12 hydrogen atoms (darker, larger balls) within the amorphous cell of pure silicon is shown. See Table 1 in Section 4.1.1.

Thus the most sensitive probe of the structure of the hydrogenated sample is, as one expects, the H-H pRDF. In Figure 7 we pictorially show the locations within the cell where the hydrogen atoms were placed at the outset of the simulation. They correspond to the coordinates given in Table 1.

#### 4.1.3. Summary

Hydrogenated amorphous silicon was the first non-single, diatomic, semiconducting material that we amorphized with the *undermelt-quench* approach and the code Fast. This computational protocol generated amorphous samples with fewer dangling bonds and fewer overcoordinated defects than the standard, widely used, *melt-and-quench* protocol; it also generated structures having total and partial radial distribution functions whose main features lined up well with experimental measurements. In the hydrogenated samples, the hydrogen atoms were not placed in position as has sometimes been done, but were allowed to move throughout the cell and quench into low energy configurations. Where dangling bonds were available, the hydrogens tended to attach there. In the absence of a sufficient defect concentration, di-hydrogen formed.

### 4.2. Amorphous Silicon-Nitrogen Alloys [1,51,52,53,54,55]

Amorphous silicon nitrogen alloys, a-SiN_x_, have attracted a great deal of attention in the last decades since they have electrical, optical, and mechanical features useful from the application viewpoint and their accentuated covalency makes them very interesting from the fundamental point of view.

#### 4.2.1. Preamble

The experimental and theoretical knowledge of the atomic, electronic, and optical properties of *a-SiN_x_* is not as ample as its importance merits. For example, experimentally their total RDFs are practically unknown, except for the stoichiometric composition, and the partial radial features are nonexistent. Theoretically, no *ab initio* attempt at simulating total and pRDFs had been performed before our work, although first-principles studies of the electronic properties of random structures *classically* generated with Tersoff-like interatomic potentials had been carried out [56]. Since no first-principles generation of random networks simulating realistic total and pRDFs had been reported, an *ab initio* approach that could adequately describe and predict the atomic topology of *a*-SiN*_x_* would be useful to understand their electronic features and to explore its applicability to other covalent amorphous diatomic materials. This is what we did several years ago.

Since experimental RDFs are scarce [57,58,59,60] and, to our knowledge, only a total RDF for the stoichiometric amorphous composition exists [61,62,63] it was not possible to obtain upper and lower experimental bounds to compare with our simulational results [1]. A Gaussian-based decomposition of the second peak of the total stoichiometric RDF into its partial contributions was carried out by Misawa *et al.* [62], and this will be the subject of comparison with our simulations.

We applied our computational protocol to 13 samples each with 64 atoms total and with different nitrogen contents *x* where 0 ≤ *x* ≤ 1.29, or where 0 ≤ (number of N atoms) ≤ 36, and performed two runs for each sample. The content is defined as *x* = *y*/(64 − *y*) where *y* is the number of randomly substituted nitrogens. The core was taken as full which means that an all electron calculation was carried out, and a minimal basis set of atomic orbitals was chosen with a cutoff radius of 5 Å for the amorphization and 3 Å for the optimization. The physical masses of nitrogen and silicon were always used and this allowed us to see realistic randomizing processes of the nitrogen atoms during the amorphization of the supercell. The default time step is 1.73 fs; however, in order to better simulate the dynamical processes that occur in the amorphization and to optimize computer resources, a time step of approximately four times the default, 6 fs, was used for all samples.

**Table 2:** Characteristics of 13 samples of amorphous silicon nitrogen alloys, each with 64 atoms total and with different nitrogen contents where 0 ≤ (number of N atoms) ≤ 36. The content is *x* = *y*/(64 − *y*) where *y* is the number of randomly substituted nitrogens.

Contents, amorphization temperatures, densities and gaps for *a*-SiN*_x_*				
Sample	*x*	Amorphous temp. (K)	Density (g/cm^3^)	Average Gap (eV)
Si64N0	0.000	1680	2.329	1.09
Si59N5	0.085	1747	2.435	1.11
Si54N10	0.185	1814	2.512	1.05
Si49N15	0.306	1881	2.600	1.19
Si44N20	0.455	1948	2.694	1.69
Si39N25	0.641	2015	2.803	2.49
Si34N30	0.882	2082	2.931	3.41
Si33N31	0.939	2095	2.957	3.73
Si32N32	1.000	2108	2.988	4.10
Si31N33	1.065	2122	3.017	4.31
Si30N34	1.133	2136	3.048	4.28
Si29N35	1.207	2149	3.081	4.69
Si28N36	1.286	2162	3.115	4.95

To avoid quenching from the melt we amorphized the crystalline diamond-like structures by linearly heating them from room temperature to just below the corresponding melting point for each *x* in 100 steps, and then cooling them down to 0 K using Fast. To determine the melting temperature for each concentration we linearly interpolated between pure silicon and the stoichiometric compound, *i.e.*, the sample with 56% nitrogen. We remained a few degrees below these values (see Table 2) and this process was then followed by cycles of annealing and quenching at temperatures suggested by experiment; finally the structures were energy optimized. Since the 6 fs time step was kept constant for all runs and the amorphization temperatures increased with *x* as indicated in Table 2, the heating/cooling rates varied from 2.30 × 10^15^ K/s for pure silicon to 3.11 × 10^15^ K/s for *x* = 1.29. The atoms were allowed to move within each cell, with periodic boundary conditions, whose volume was determined by the corresponding density and content. The densities were taken from the experimental results of Guraya *et al.* [59] (See Table 2). Once this first stage was completed, Fast was used to subject each cell to annealing cycles at 300 K with intermediate quenching down to 0 K. Finally, the samples were energy optimized to make sure that the final structures would have local energy minima.

#### 4.2.2. Results and Analysis

**Figure 8:**
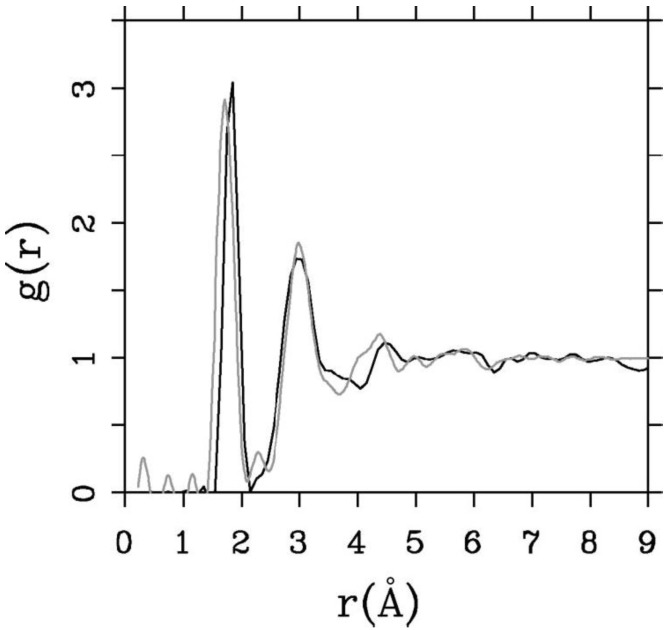
RDFs for silicon nitride, the stoichiometric. The lighter line is the experimental curve. The darker line is our two averaged results (*a*-SiN_1.29_), properly weighted, with ρ = 3.115 g/cm^3^, a 6 fs time step, and a 5 Å cutoff [55].

Since the stoichiometric compound is the one that has been experimentally studied [61,62,63] we compared our averaged simulated results for this alloy to the experimental data. In Figure 8 it can be seen that the agreement is very good.

In addition, Table 3 gives some parameters for *a*-SiN_1.29_. This Table shows the positions and maximum values of the first and second peaks, and the average number of nearest neighbors <*nn*>. The minimum between the first and the second peaks is 2.15 Å and this value was used to obtain <*nn*>.

**Table 3:** Topological parameters for the RDF of the stoichiometric SiN amorphous alloy.

Height and position of the maxima of radial peaks and nearest neighbors <*nn*> for *a*-silicon nitride (*x* = 1.29)					
Material	FIRST PEAK		SECOND PEAK		<*nn*>
	Position	Height	Position	Height	
SiN_1.29_	1.85 Å	3.04	2.95 Å	1.73	3.47

**Figure 9:**
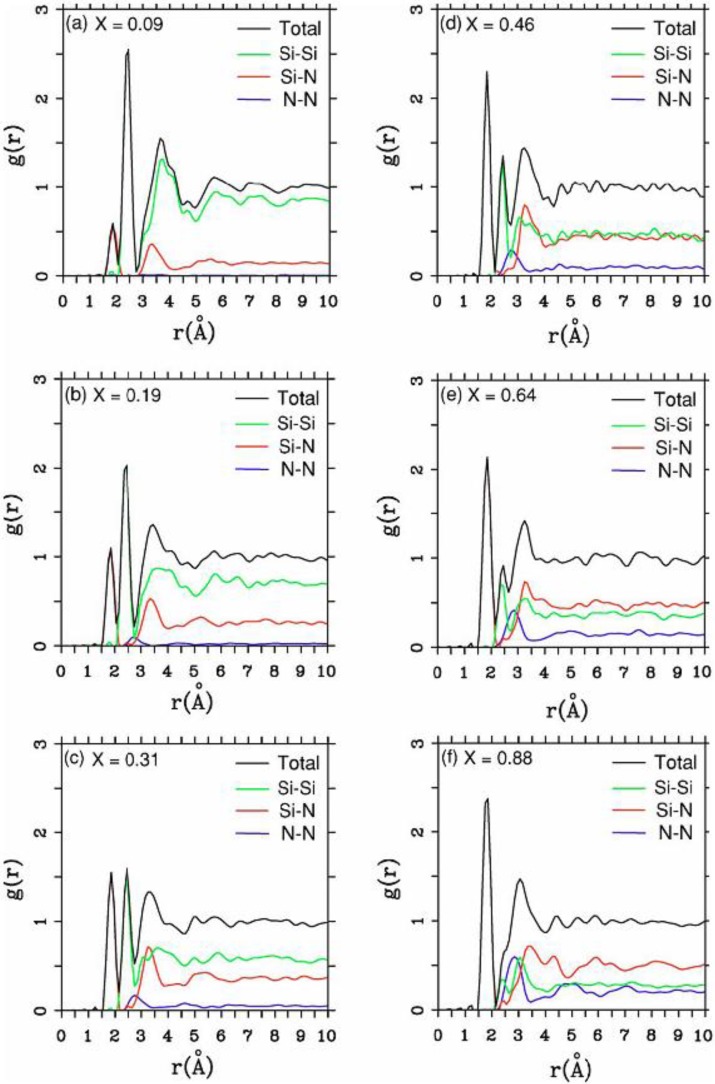

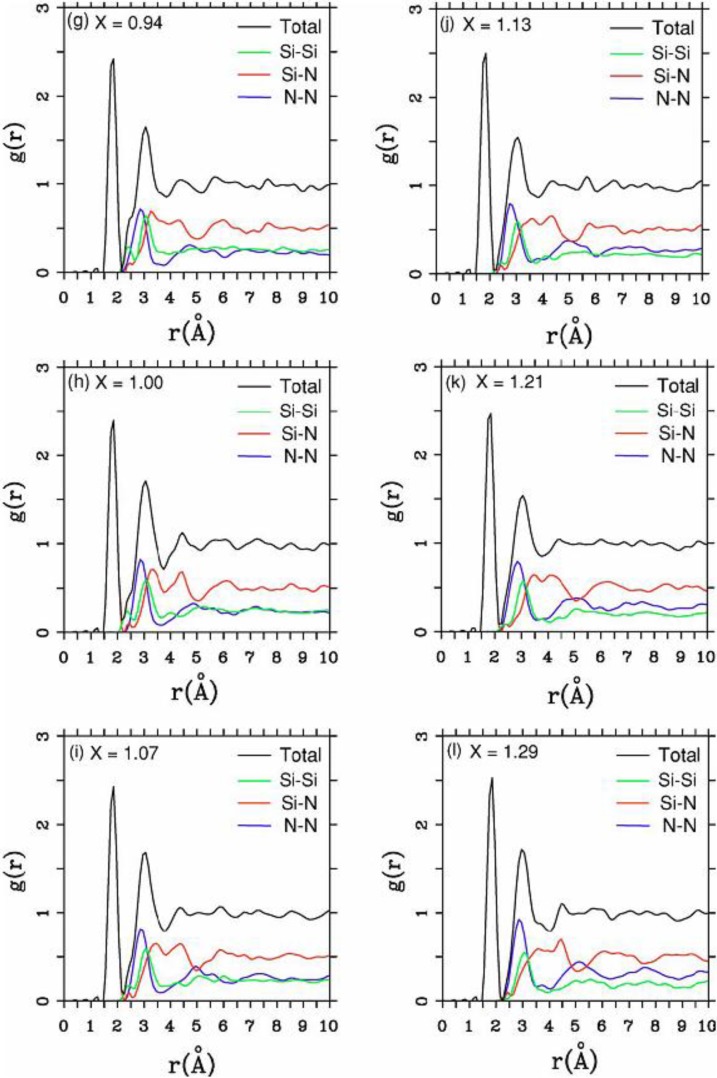
Total and partial RDFs for *a*-SiN*_x_*. The contents are: (**a**) *x* = 0.09; (**b**) *x* = 0.19; (**c**) *x* = 0.31; (**d**) *x* = 0.46; (**e**) *x* = 0.64; (**f**) *x* = 0.88; (**g**) *x* = 0.94; (**h**) *x* = 1.00; (**i**) *x* = 1.07; (**j**) *x* = 1.13; (**k**) *x* = 1.21 and (**l**) *x* = 1.29 (the stoichiometric).

This gave us confidence to be predictive with respect to other concentrations of nitrogen, and total and partial radial features are presented for a variety of nitrogen contents in Figure 9 where we report 12 total and partial RDFs for the corresponding non-zero contents of nitrogen that were used in the simulations. Hopefully such graphs will invite experimentalists to produce and study samples with nitrogen concentrations different from the stoichiometric material. The contents are: (a) *x* = 0.09, (b) *x* = 0.19, (c) *x* = 0.31, (d) *x* = 0.46, (e) *x* = 0.64, (f) *x* = 0.88, (g) *x* = 0.94, (h) *x* = 1.00, (i) *x* = 1.07, (j) *x* = 1.13, (k) *x* = 1.21 and (l) *x* = 1.29 (the stoichiometric).

As the nitrogen content increases the first peak of the total RDF (1.85 Å) which is due to the Si–N average nearest-neighbor contributions <*nn*> increases systematically and the <*nn*> Si–Si peak (2.45 Å) decreases systematically. The third peak of the total RDFs moves toward lower *r*, 3.25 Å to 2.95 Å, as *x* increases since the N–N contribution becomes more predominant for higher content. In our structures there are no <*nn*> nitrogens since the content is below stoichiometry and nitrogens have a marked tendency to bind to silicons. No di-nitrogen is formed even though for *x* > 1 the starting diamond structure *does* contain nearest-neighbor nitrogens. For the nearly stoichiometric sample (*x* = 1.29), Figure 9(l), the Si–Si <*nn*> contribution to the total RDF has practically disappeared. This implies that there is a nitrogen atom between every pair of silicons indicating a tendency to form 6-atom closed rings, Si–N–Si–N–Si–N, typical of the Si_3_N_4_ structures. The growth of the Si–N peak as nitrogen increases bears out this behavior.

The curves of Figure 9 are bare RDFs and represent the number of atoms at a certain radial distance from a given one. In order to compare with X-ray or neutron diffraction experiments the corresponding diffraction weights must be calculated, applied to the partial contributions, and then summed to give the total RDFs. This is what we did for the stoichiometric sample, Figure 8.

In order to *quantitatively* compare our predictions, presented in Figure 9, with experiments one needs to use the expression quoted by Aiyama *et al.* [61]:

*g(r)* = [*c*_1_^2^*f*_1_^2^ ∕<*f*>^2^] *g*_11_(*r*) + 2[*c*_1_*c*_2_*f*_1_*f*_2_ ∕<*f*>^2^] *g*_12_(*r*) + [*c*_2_^2^*f*_2_^2^∕<*f*>^2^] *g*_22_(*r*)

where *c*_1_ is the ratio of the number of silicon atoms to the total number of atoms and *c*_2_ the ratio of the number of nitrogen atoms to the total number of atoms. *f*_1_ is the silicon structure factor for X-ray scattering, or the scattering amplitude for neutron scattering, and *f*_2_ is the factor, or the scattering amplitude, for nitrogen. *gij*(*r*) are the partial pair distribution functions, *g*_11_ = *g_SiSi_*, *etc*. Finally, <*f*>^2^ = (*c*_1_ *f*_1_ + *c*_2_ *f*_2_)^2^.

It is understandable that silicons are more prominent for X-rays than for neutrons, and the opposite occurs for nitrogens. That is why [(*c_Si_*^2^ *f_Si_*^2^)/<*f*>^2^] *g_SiSi_* (*r*) is more prominent for the X-ray simulation and [(*c_N_*^2^ *f_N_*^2^)/<*f*>^2^] *g_NN_* (*r*) is more prominent for the neutron simulation. This observation is responsible for the displacement of the position of the maximum of the second peak of the stoichiometric total radial distribution function, as shall be seen later on in this section.

**Figure 10:**
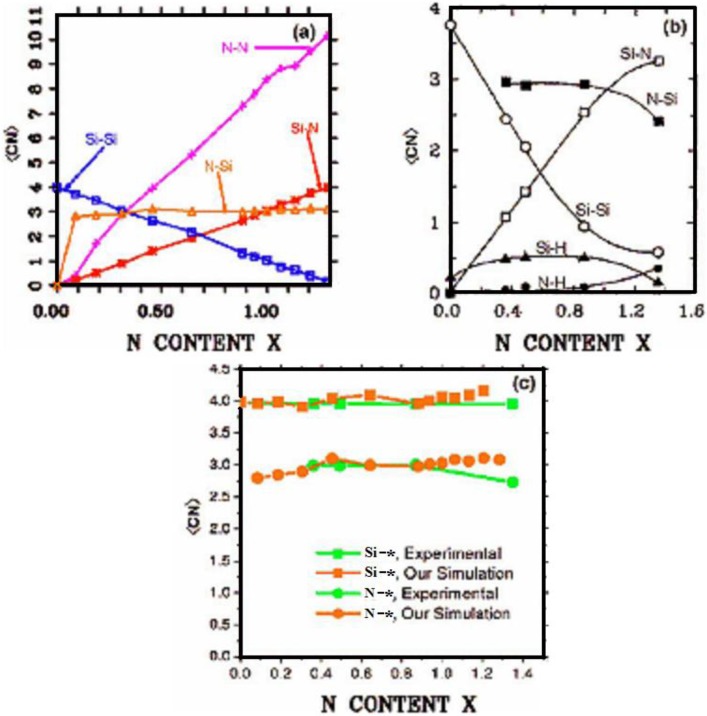
Average coordination numbers <*cn*> as a function of *x*. (**a**) Our results; (**b**) Experimental results for *hydrogenated* alloys from Guraya *et al.* [59]; (**c**) Comparison of the integrated results (see text). Lines are drawn as guides to the eye.

In Figure 10 a study of the average coordination numbers <*cn*> in the (12 + 1) random networks is depicted. Based on the bonding assumption discussed at the end of Section 3, the following cutoff radii were used: Si–Si, 2.55 Å; N–N, 3.35 Å and Si–N, 2.15 Å, which are the positions of the minima after the first peaks of the corresponding pRDFs. Figure 10(a) shows the results of our simulations and it can be seen that the N–N plot has a plateau for *x* ≥ 1.1, the percolation threshold of Si–Si bonds. The number of Si-Si <*nn*> goes from 4 to practically 0. The Si–N graph refers to the <*nn*> nitrogens around the silicon atoms and varies from zero to four, whereas the N–Si refers to the <*nn*> silicons around nitrogens and indicates that nitrogens immediately surround themselves with practically three Si, saturating its valence. There is a crossing of the Si–Si, N–Si, and N–N plots at *x* ≈ 0.3 and a crossing of N–Si and Si–N at *x* ≈ 1.0 which have been observed experimentally for *hydrogenated* alloys by Guraya *et al.* [59], Figure 10(b).

However, due to the presence of hydrogen a curvature appears in the <*cn*> for Si–Si, Si–N, and N–Si, so in order to compare our results with this experiment, we did the following. We calculated the sum of N–H and N–Si from the experiment, the average total number of atoms that surround a N:N–*, and plotted it along with our N–Si. We also did the sum of the experimental Si–N, Si–Si, and Si–H, the average total number of atoms that surround a Si:Si–*, and plotted that along with our sum of Si–N plus Si–Si. This is presented in Figure 10(c). It is clear that our predictions closely agree with the integrated experimental results and show that our structures are realistic. The discrepancies are most likely due to existing dangling and floating bonds. See Reference [55] for a more ample and complete discussion.

**Figure 11:**
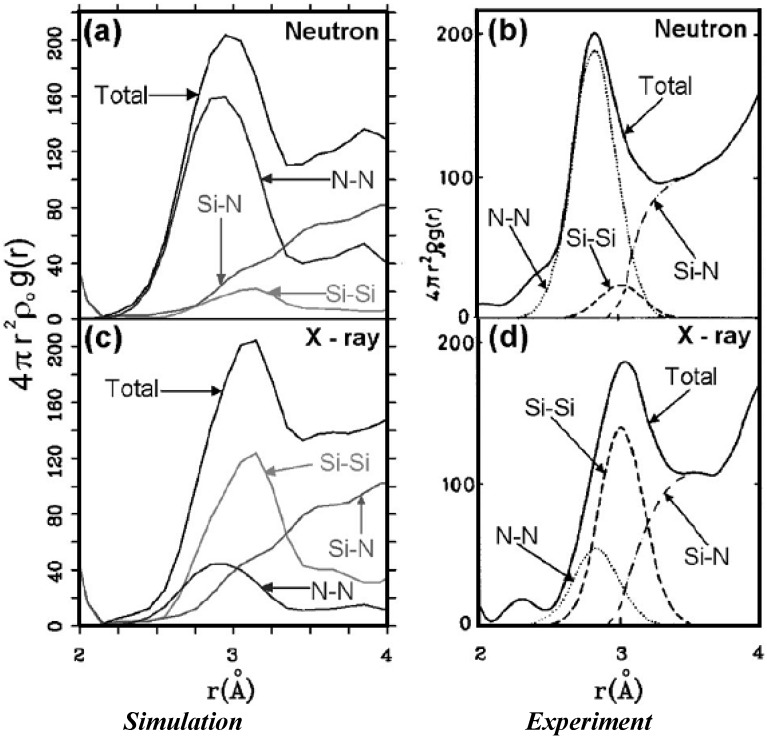
Comparison of the simulated (**a**) and (**c**), and experimental (**b**) and (**d**), structures of the second peak of the nearly stoichiometric sample when neutrons and X-rays are used, respectively. For this peak X-rays show a shift toward higher values of *r* with respect to neutrons, which is reproduced in our simulations.

The composition of the second peak of the total RDF for the stoichiometric sample is given in Figure 9(l) and it agrees *qualitatively* with experiment by Misawa *et al.* [62], since it is formed by the average second neighbor (<2*n*>) contributions of mainly the N–N and Si–Si partials and to a lesser extent the Si–N partial [54]. In order to *quantitatively* compare our predictions with the few experiments available we calculated the structure of the second peak of the nearly stoichiometric cell considering whether the radial features were determined using X-rays or neutron diffraction experiments. Figure 11 shows the experimental and simulated results and the *quantitative* agreement is excellent even though the experimentalists only used Gaussian fits to simulate the structure of the second peak. For this peak X-rays show a shift of the total RDF toward higher values of *r*, with respect to the neutron results, which is reproduced in our simulations [54,55].

**Figure 12:**
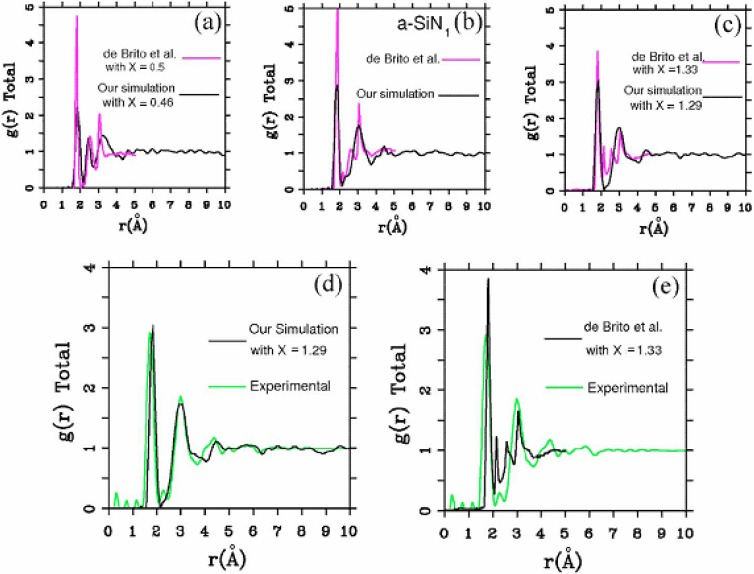
Comparison of our results and those obtained by De Brito Mota *et al.* [64,65,66,67] for the total RDF. The agreement is at best qualitative; the position of some of the peaks coincide for the samples (**a**)–(**c**). The experimental total RDF (green curve) is compared to our simulations, (**d**) and to de Brito Mota’s simulations (**e**) (black curves).

Finally, Figure 12 is the comparison of our *ab initio* results with the classical Monte Carlo simulations of De Brito Mota *et al.* [64,65,66,67] who used empirical potentials developed *a la* Tersoff for the interactions between Si and N. It is clear that although the positions of some peaks are reproduced in both simulations, the total RDFs do not agree. Comparison of the two simulations with experiment for the stoichiometric samples is also presented in this figure, where the discrepancies of the classical approach with experiment clearly show the difficulties involved in constructing parameterized classical potentials to describe binary systems that are basically quantum mechanical.

#### 4.2.3. Summary

Evidently our *ab initio* simulations of *a*-SiN*_x_* (0 ≤ *x* ≤ 1.29) are very successful. To our knowledge there are no other simulations, classical or quantum-mechanical, that lead to atomic topologies in agreement with existing experimental data to this extent. The atomic structures have total RDFs and average coordination numbers that agree with the experimental results available. The simulated pRDFs show that the Si–Si <*nn*> peak disappears as nitrogen increases indicating a tendency to form 6-atom arrangements as the content *x* approaches the stoichiometric value. Experiment shows that for a-Si_3_N_4_ Si and N form 6-atom closed rings, Si–N–Si–N–Si–N, typical of the crystalline Si_3_N_4_ structures. The growth of the Si–N peak as nitrogen increases bears this out. For *x* ≥ 1.1 the effects of the percolation threshold of the Si–Si bonds is observed in the N-N <2*n*> and in the optical gaps as we shall see later (Section 5). For *x* ≈ 0.7 the Si–Si and Si–N neighbors are practically the same, as found experimentally. Also, Si–Si, N–Si, and N–N are practically the same for *x* ≈ 0.3 as are Si–N and N–Si for *x* ≈ 1.0. The integrated experimental results for <*cn*> and our simulations agree. An analysis of the second peak in the total RDF of the nearly stoichiometric sample indicates that N–N, Si–Si, and Si–N contribute to it, in qualitative agreement with experiment. However, when the partials are weighted, the agreement becomes quantitative. We report all the bare pRDFs and total RDFs for the contents studied; these curves have to be weighted accordingly to be compared with experiment.

### 4.3. Amorphous Carbon-Nitrogen Alloys [1,68]

It is well known that carbon is a versatile element due to the variety of hybridization states that it assumes which lead to a multiplicity of bond arrangements fostering the formation of compounds with varied structures. This versatility is reflected in its amorphous phase which, depending on its density, may have atomic arrangements that go from polymeric-like to diamond-like, including graphitic structures; that is, the study of amorphous carbon is a density dependent issue [69,70,71]. Nitrogen also manifests several possible bonding arrangements, and when it is incorporated into amorphous carbon the variety of structures that can be formed is evidently much larger [72], exhibiting up to 9 different competing forms. It is then clear that the description of the bonding nature of the amorphous carbon-nitrogen system can be highly complex, and the unambiguous identification of the bonding types is difficult. In addition, the experimental density of the CN system depends drastically on the specific process used to generate the material, and not just on the nitrogen concentration, to the point that the dispersion in the experimental correlation between density and concentration is large and trying to find a systematic behavior becomes difficult [73].

#### 4.3.1. Preamble

There is a need to understand amorphous CN since it is potentially useful in diverse applications. With nitrogen incorporation, amorphous carbon changes its electrical and optical properties [74,75,76,77]. It is also important to study the effect that the incorporation of nitrogen into an amorphous carbon matrix has on the bonding structure. This knowledge would allow one to tailor these structures to display specific properties. It is experimentally known that resistivities and optical band gaps decrease, compared to the pure tetrahedral *a-*C films, *ta-*C, when nitrogen concentration increases [74,75,76,77]. Despite the difficulties encountered with bonding characterization, two other experimental observations make this system attractive: one has to do with the existence of a real upper limit found for the amount of nitrogen that can be dissolved within a carbon matrix; the other is related to the behavior of nitrogen as a possible dopant at low concentrations. It has been established that it is impossible to generate amorphous CN systems with concentrations above 20%–30% nitrogen since this element simply escapes from the material at atmospheric pressure [78,79,80]. Also, it has been reported experimentally that for concentrations less than 5%, nitrogen increases the conductivity of amorphous carbon in a manner similar to the conventional *n*-type doping of crystalline silicon; that is, nitrogen becomes a donor [81,82].

As mentioned in Section 2, *ab initio* CPMD studies of this system are scarce. In recent work by Merchant *et al.* random networks were generated by melting a crystalline 64-atom supercell and quenching it afterwards [16]. They used Wannier function techniques contained in the CPMD approach to describe the types of bonding, and also studied the eDOS to investigate the doping mechanism of carbon by nitrogen. Only two concentrations were considered, each for three different densities. The two concentrations were C_62_N_2_ and C_56_N_8_, and the three densities were 2.45 g/cm^3^, 2.95 g/cm^3^, and 3.20 g/cm^3^. An additional simulation was carried out for a density of 2.7 g/cm^3^ and a concentration of C_60_N_4_. Because of the small number of atoms in the supercell (64) and the few concentrations considered, it is difficult to see tendencies and the systematics of their results. In previous studies by this group [17] they found that when nitrogen was substituted for an *sp*^2^-bonded carbon atom, the site remained as three-fold coordinated, and a lone-pair orbital developed. When nitrogen was substituted for an *sp*^3^*-*bonded carbon atom, a bond was broken and the coordination of the site was reduced to 3. The fourth bond was lengthened to accommodate the lone-pair orbital on the nitrogen atom. They claimed that, contrary to the substitutional doping experimentally found, their results did *not* indicate the presence of tetrahedral nitrogen. With respect to the other experimental fact mentioned above, it is clear that due to the low concentrations of N used in both papers, the detection of some signal concerning the limit of doping with nitrogen would have been difficult.

We have applied our computational protocol, based on the *undermelt-quench* approach and the code Fast, to the carbon-nitrogen system [68]. We report the *ab initio* generation of amorphous networks up to concentrations of nearly 45% nitrogen. We start with crystalline supercells with 216 atoms of carbon and nitrogen and to avoid unnecessary parameters, the densities used are the experimental values fitted to a straight line; we generate the amorphization by heating them to a value below the melting temperature (*undermelt*). After this undermelting process we *quench* the samples.

Our aim, in what follows, is to show that our results can reproduce the two experimental facts mentioned above. We also find that the variation of *n*-fold coordinated nitrogen as a function of concentration agrees with several experiments. These results are based on what could be an assumption applicable to other covalent systems, *i.e.*, the assumption established at the end of Section 3: the geometrical fact that the bond length in these amorphous systems can be taken as the value of the first minimum of the corresponding pRDF, especially when this minimum is zero. We have been consistently using this assumption in our studies of binary covalent systems and the results obtained support the approximation.

In the cubic diamond-like supercells of 216 atoms, (216 − *y*) are carbons, *y* are randomly substituted nitrogens and the concentration is *c* = (*y*/216) × 100. The supercells are linearly heated, using Fast, from 300 K to a value below the melting temperature, in 100 steps of 4 fs and immediately cooled down to 0 K in 108 steps. A process like this has been applied to carbon [83] where a high temperature of 3,700 K was used obtaining RDFs very close to experiment. Due to the lack of information concerning the melting temperatures of the CN system we decided to use the value of 3,700 K for all samples [83], which is below the melting temperature of pure carbon. Since the time step (4 fs) and the melting temperature (3,700 K) were kept constant, the heating/cooling rate was also constant, 8.50 × 10^15^ K/s. The atoms were allowed to move within each cell with periodic boundary conditions, and the cell volumes were determined by the corresponding experimental densities and concentrations. We next subjected them to annealing cycles at 300 K, with intermediate quenching steps down to 0 K. At the end of the process a geometry optimization was carried out to find the amorphous structures in a local energy minimum. The densities considered were obtained from a linear fit adjusted to the experimental data: ρ = (2.897 − 1.784*c*) g/cm^3^, where *c* is the nitrogen concentration; the density values for each of the 9 nitrogenated samples, plus the pure carbon sample, are listed in Table 4.

**Table 4:** Supercells of the amorphous alloy CN with the nitrogen concentrations and their densities.

Concentrations and densities for *a*-CN		
Sample	N concentration *c* (%)	Density **ρ = [2.897 − 1.784*c*]** (g/cm^3^)
C216N00	0.0	2.90
C205N11	5.1	2.81
C194N22	10.2	2.72
C184N32	14.8	2.63
C173N43	19.9	2.54
C162N54	25.0	2.45
C151N65	30.1	2.36
C140N76	35.2	2.27
C130N86	39.8	2.19
C119N97	44.9	2.10

Because carbon and nitrogen have such a small number of electrons, an all electron calculation was carried out. We used a minimal basis set, consisting of the atomic orbitals occupied in the neutral atom, with a cutoff radius of 3.5 Å for the amorphization and for the optimization. For each atom, one function is used to represent the core part of the electron density and one to represent the valence part of the electron density. The physical masses of carbon and nitrogen are always used and this allows the visualization of realistic randomizing processes of all the atoms during the amorphization of the supercell.

#### 4.3.2. Results and Analysis

Based on the bonding assumption discussed at the end of Section 3, we proceeded to obtain the number of nearest neighbors to any carbon or any nitrogen as a function of nitrogen concentration using the fact that the first minima of the corresponding pRDFs are the same for all concentrations and are given by 2.0 Å for C–C, 1.9 Å for C–N, and 1.8 Å for N–N.

We focused mainly on the nearest neighbors to nitrogens, in order to investigate the two experimental results mentioned above; namely, the fact that nitrogen becomes tetrahedrally coordinated at low concentrations and the fact that there is an experimental upper limit to the nitrogen concentration in an amorphous carbon matrix. Once the maxima of the bond lengths are set, then the number of nitrogen atoms that are single-fold, two-fold, three-fold, and four-fold coordinated can be determined; no five-fold or higher bonding arrangements were found.

**Figure 13:**
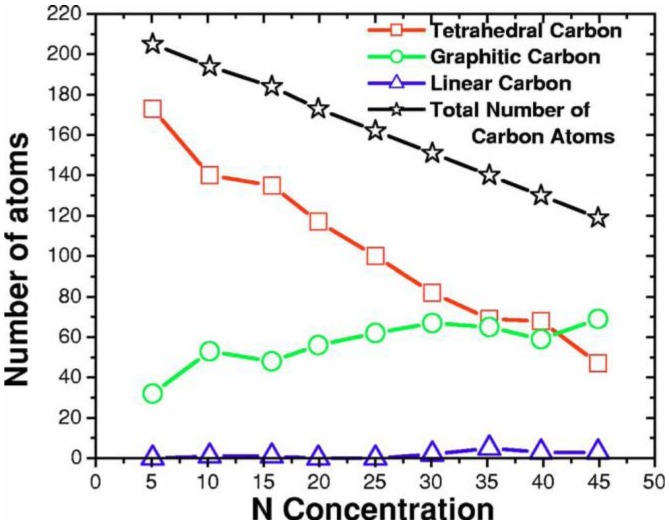
The bonding nature of carbon is shown as a function of nitrogen concentration. The tetrahedral carbons (squares) decrease whereas the graphitic carbons (circles) increase as the nitrogen concentration increases.

In Figure 13 the dependence of the bonding nature of carbon with nitrogen concentration is presented. Evidently, the total number of carbon atoms diminishes linearly as the number of nitrogens increases, and there is an almost linear variation of tetrahedral and graphitic carbon. Tetrahedral (four-fold) carbons show a plateau between 10% and 15% and another between 35% and 40%, although this may be the result of statistical fluctuations and larger cells should be studied. Linear (two-fold) carbons begin appearing at 30% and graphitic (three-fold) carbons behave in a complementary manner.

**Figure 14:**
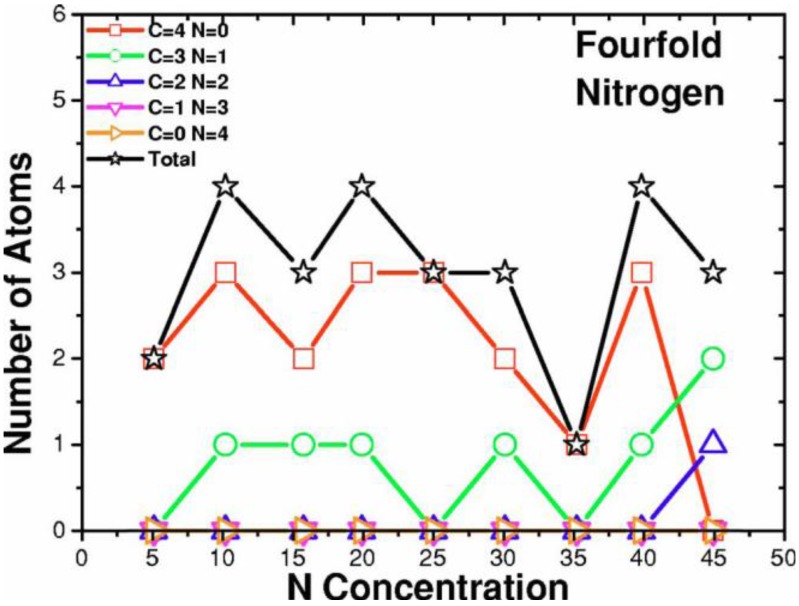
Tetrahedral (dopant) nitrogens are prominent at low concentrations, 0–10%. This behavior is masked by other types of bonding for higher concentrations. Bonding to 4 carbons is represented by squares; to 3 carbons and one nitrogen by circles.

No five-fold coordinated carbons were found. At about 15% nitrogen, graphitic carbons diminish and, correspondingly, tetrahedral carbons increase; at 35% the number of graphitic and tetrahedral carbons is practically the same, whereas linear carbons become nonzero. For concentrations larger than 40%, graphitic carbons outnumber tetrahedral carbons and linear carbons are distinctively finite. This behavior indicates a slight departure from linearity at about 10% and 35%; this departure could be related to the behavior of nitrogen as a dopant below 10% and to the saturation limit around 35%; clearly more information is needed to support this point.

In order to further investigate the bonding structure of these alloys we looked at the possibility of finding four-fold (overcoordinated) nitrogens for certain concentrations. In fact, at 5% (the lowest concentration of nitrogen we investigated) the first sign of overcoordination appears and this is due to the coordination of one nitrogen atom with four carbons, Figure 14. Such overcoordination should manifest itself at concentrations lower than 5% if continuity in the behavior occurs. This four-fold coordination leads to the appearance of an electronic state within the energy gap [84].

**Figure 15:**
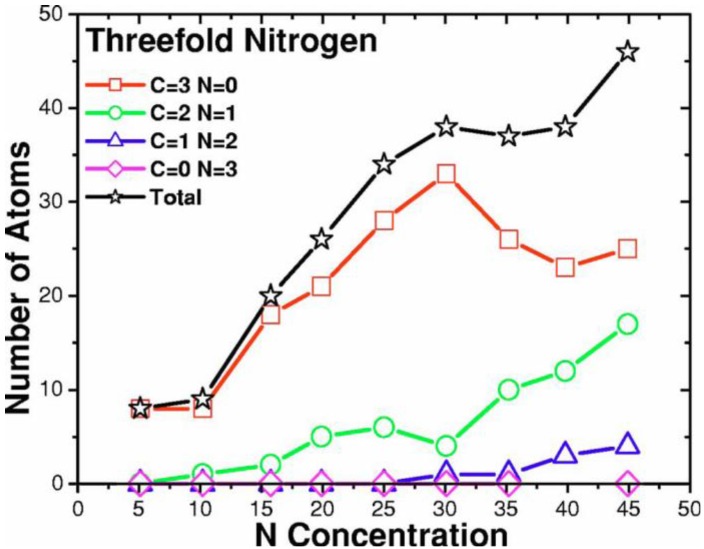
Nitrogens triple bonded. The total number is practically constant between 5% and 10%. The squares are nitrogens bonded to three carbons; the circles are nitrogens bonded to two carbons and one nitrogen.

**Figure 16:**
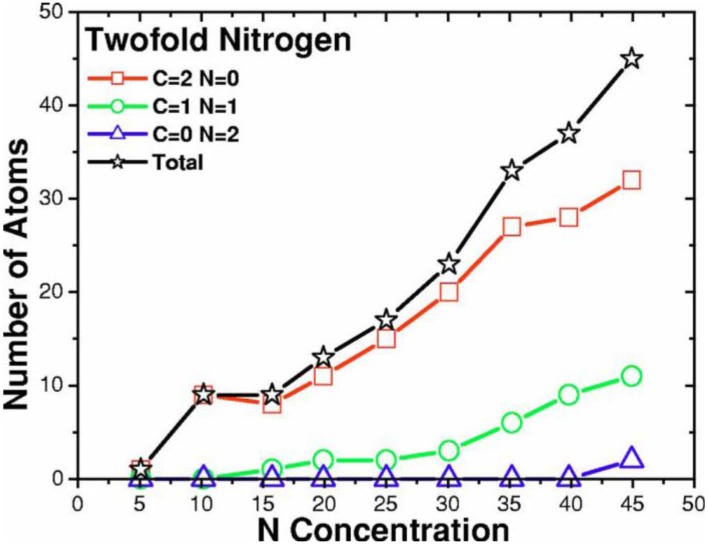
Nitrogen double bonded. The total number is practically zero at 5%, shows a plateau between 10% and 15% and increases systematically afterwards. Squares represent nitrogens bonded to two carbons and circles to one carbon and one nitrogen.

It is interesting to note that four-fold coordination due to three carbons and one nitrogen atom is also important up to 35% nitrogen concentration. As the number of nitrogen atoms increases the preponderance of these four-fold-coordinated atoms becomes less relevant since they remain constant while other types of bonding become more prominent. See total curves in Figure 15 and Figure 16.

Figure 15 shows how a three-fold coordination of nitrogen with three carbons appears at 5% and increases up to 30%. Thereafter it decreases while an important rise of the coordination with two carbons and one nitrogen begins. Coordination with one carbon and two nitrogens starts growing at 25%, while three-fold coordination with three nitrogens never appears in the range studied.

It is clear that something drastic happens at 30% since the three-fold coordination with three carbons starts diminishing abruptly, Figure 15. We believe this is another indication of the existence of an upper limit to the nitrogen incorporation into an amorphous carbon matrix. Double bonded nitrogen appears at larger concentrations (10%) and continuously increases up to 45%, Figure 16. A plateau is observed between 10% and 15% for the total two-fold coordination and for nitrogen bonded to two carbons. Double bonded nitrogen to one carbon and one nitrogen increases slowly but is always smaller than the previous case. Two-fold coordination to two nitrogens is nonexistent except at the highest concentration, which incidentally may not exist as a stable solid amorphous system.

**Figure 17:**
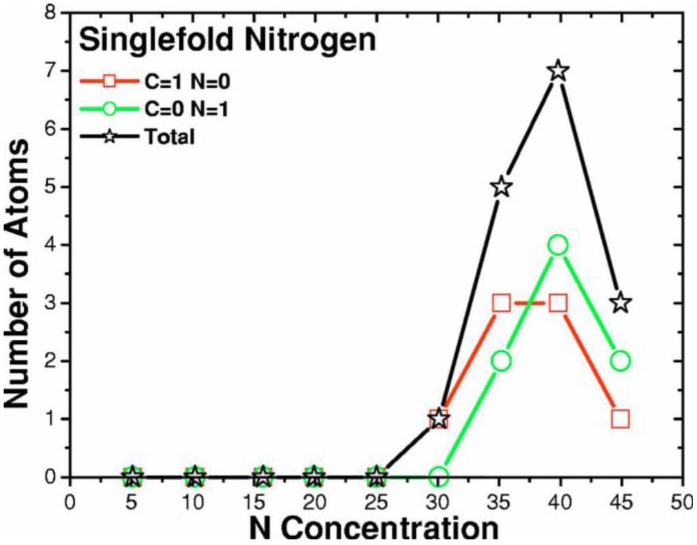
Single bonded nitrogens appear only at concentrations larger than 25%. There are nitrogens bonded to one carbon (squares) and to another nitrogen, molecular nitrogen (circles).

Figure 17 shows that single bonded (single-fold) nitrogen coordinated to one carbon exists starting at 25%; it has a maximum at about 35%–40% and then begins to decrease. Something similar occurs with the appearance of N–N coordination since it begins at 30%, two single-fold nitrogen atoms appear at 35%, reach a maximum of four at 40%, and then diminish. This behavior indicates the possible formation of nitrogen molecules N_2_ in the system. But why should we have molecular nitrogen in these amorphous alloys for large concentrations? We believe that since our approach does not allow nitrogens to abandon the cubic supercell when a saturation concentration is reached, the nitrogen cannot leave the material and therefore remains in the gaseous state within the CN system; eventually it forms molecules that appear in the voids of the supercell.

**Figure 18:**
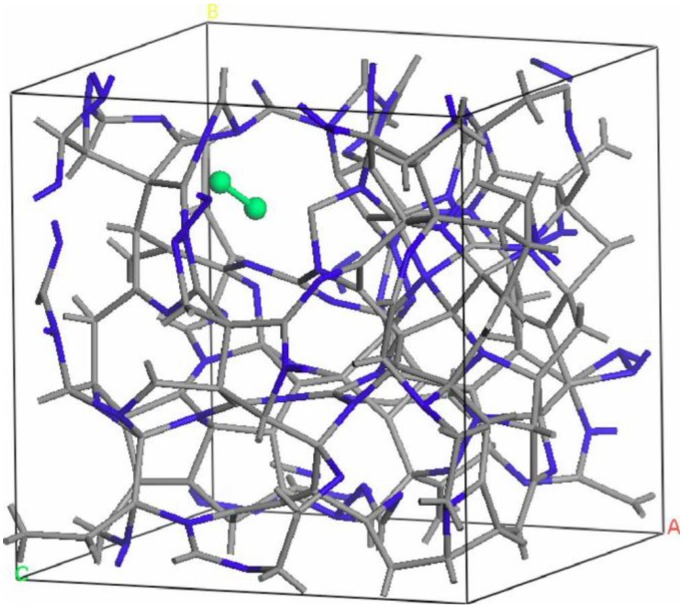
For concentrations above 25% single bonded nitrogen exists. At 35% a nitrogen molecule (green dumbbell) appears signaling the saturation limit to nitrogen incorporation. Carbon: light gray sticks; nitrogen: blue sticks.

**Figure 19:**
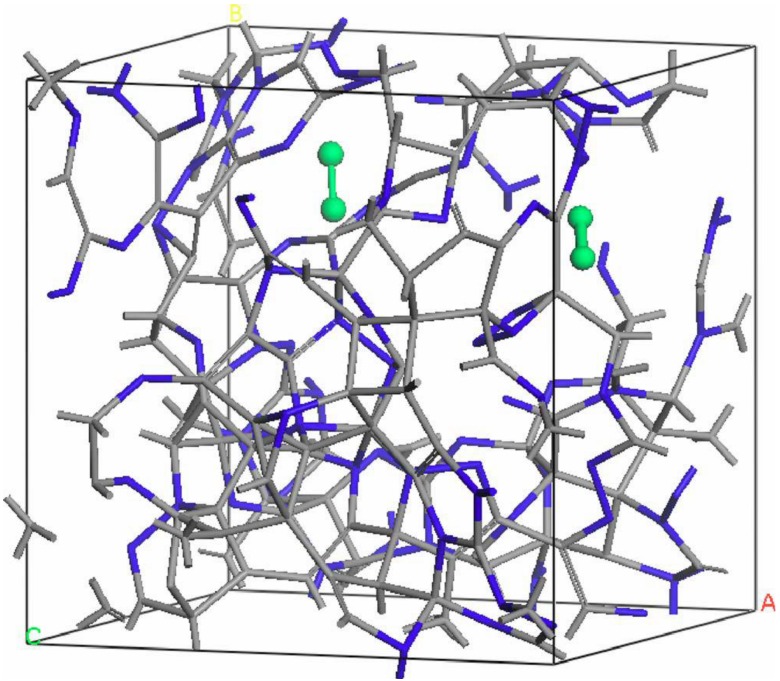
At a concentration of 39.8% two nitrogen molecules (green dumbbells) appear, indicating that we are already within the saturation region. Carbon: light gray sticks; nitrogen: blue sticks.

**Figure 20:**
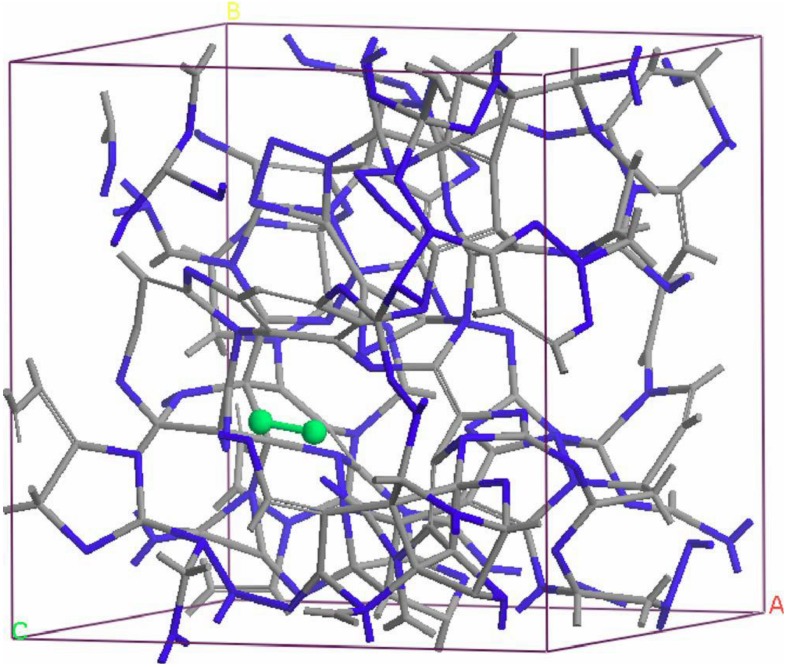
At a concentration of 44.9% of nitrogen, N_2_ molecules still exist (green dumbbell), well within the saturation region. Light gray sticks correspond to carbon, blue sticks correspond to nitrogens.

This explanation is explicitly corroborated in Figure 18, Figure 19 and Figure 20, where the molecules are shown: one molecule (two nitrogen atoms) for 35% (1.26 Å interatomic distance); two molecules (four nitrogen atoms) for 40% (1.25 Å and 1.26 Å), and one molecule (two nitrogen atoms) for 45% (1.25 Å). It is clear then that this phenomenon signals the presence of a saturation limit in the CN system.

#### 4.3.3. Summary

Carbon bonding is versatile; conventionally, three different types of hybridizations are attributed to carbon, four-fold *sp*^3^, three-fold *sp*^2^, and two-fold *sp*^1^ with precise orientations. In amorphous materials with carbon content it is difficult to talk about the orientation of bonds since the resulting structure is not geometrically well defined; nevertheless these three hybridizations are invoked as a basis for the analysis of the atomic topology. Nitrogen bonding is also versatile since nitrogen has five valence electrons and it also manifests three different types of hybridizations. However, the electronic arrangement is somewhat different since electron lone pairs appear. A thorough analysis of the possible bonds in the crystalline or molecular CN system leads to nine different structures. Since the orientation of bonds in an amorphous system is not well defined it is difficult to talk precisely about well defined hybridized bonds, and this complicates even more the description of the bonding structure in the amorphous CN system.

In the present work we opted for the geometric approach (Section 3) to determine the bond length and looked at the first minima of the pRDFs as a criterion for the maximum extension of the bonds between pairs of elements. This cutoff allowed us to unambiguously talk about nearest neighbors to a given atom. Based on this approach we conclude that:

(i) Tetrahedral nitrogen (*ta*-N) appears in our *ab initio* generated CN samples. The amount of *ta*-N is essentially constant and small (Figure 14) for all concentrations studied and therefore becomes more relevant for concentrations below 5% where the number of other *n*-fold coordinated nitrogens, with *n* ≤ 3, is also small. At higher concentrations *ta*-N exists but its presence is masked by all the other bonding arrangements of nitrogen (Figure 15, Figure 16 and Figure 17). These results are in agreement with the EELS experiments reported in the literature.
(i) For concentrations higher that 30%, single-fold nitrogen appears bonded to nitrogen, giving rise to N_2_ molecules (Figure 18, Figure 19 and Figure 20). We take this as signaling the onset of an upper limit for the incorporation of N in an amorphous carbon matrix, in agreement with experiment and supporting the surmise that the experimental saturation of the nitrogen content within the films is due to the formation of molecular nitrogen either at or below the film surface. Another indication that something drastic happens at 30% is that the three-fold coordination of nitrogen with three carbons starts diminishing abruptly, Figure 15.

Our results also show the essentially linear decrease of the *sp*^3^ carbon fraction with increasing nitrogen concentration reported experimentally, and the corresponding increase of the *sp*^2^ carbon fraction, Figure 13. This behavior suggests that for the densities used, carbon atoms bonded to nitrogen revert to *sp*^2^ bonding. It is then clear that unlike what happens in the SiN system, where nitrogens immediately surround themselves with practically three silicons, saturating their valence [55], in CN the situation is more versatile. We hope our results may stimulate further experimental and theoretical studies in the area.

### 4.4. Amorphous Binary Alloys Based on Group IV Elements: The Case of a-Si_0.5_C_0.5_ [85,86]

Much has been mentioned about the chemical order that may appear in these alloys, caused by heteronuclear bonds. No conclusive evidence has been reached so far, since there seems to be as many structures and atomic topologies as papers published. Pure carbon has an atomic structure in which single, double and triple bonds are important whereas in the silicon samples this multiplicity does not occur since the silicon bond is not as versatile as the carbon bond. When half the atoms are C and the other half are Si one expects that, for complete chemical order, a given Si may be surrounded by four C, and *vice versa*. Simulational results lead to a variety of reported structures that makes these alloys very interesting.

#### 4.4.1. Preamble

As mentioned in Section 2, Finocchi *et al.* [19], in 1992, performed CPMD on two different samples, one with 27 C and 27 Si atoms randomly distributed in the diamond crystalline positions and the other with 32 C and 32 Si atoms in the rock-salt crystalline structure positions. Both samples had a density of 3.1 g/cm^3^ and using the *melt-and-quench* procedure the authors heated the samples up to a temperature of approximately 4,000 K. After equilibration during 1 ps the samples were cooled down to approximately 500 K. They used a simulation time step of 4.0 a.u. for both runs and a fictitious mass parameter entering the CP equations of 300.0 a.u. They found that the two samples had very similar structural properties, and concluded that the simulations were not dependent on the initial structures. The total and partial radial distribution functions and the eDOS were reported. The RDFs had a peak around 1.5 Å due to C–C bonds and 1.9 Å due to Si-C bonds, and 40–45% of the bonds present in the sample were C–C homonuclear bonds.

In the second paper Finocchi *et al.* [20] studied the local atomic environment of *a*-Si_0.5_C_0.5_. They generated the amorphous structure of SiC using a technique very similar to their previous work [19]. In this paper, they could not establish either a chemically ordered or a completely random sample structure. They remarked that a detailed analysis of each atomic species is of vital importance in order to understand the physical and chemical properties of *a*-Si_0.5_C_0.5_.

#### 4.4.2. Results and Analysis

We used two types of Si_0.5_C_0.5_ crystalline supercells at the start, both with a density of 2.75 g/cm^3^, the experimental value. One was a diamond-like periodic supercell of 64 atoms, containing 32 carbons and 32 silicons, chemically ordered to begin with, amorphized using Fast. The other was an fcc crystalline periodic supercell with 108 atoms, 54 carbons and 54 silicons, also chemically ordered, amorphized using DMol3 from the suite in Materials Studio 3.2. The amorphization was done by heating the periodic samples from 300 K to 2,800 K for the diamond-like and to 3,053 K for the fcc, just below their melting points [87] (the *undermelt-quench* approach) and then cooling them down to 0 K. Then the structures were relaxed by annealing and quenching, and finally a geometry relaxation was carried out.

**Figure 21:**
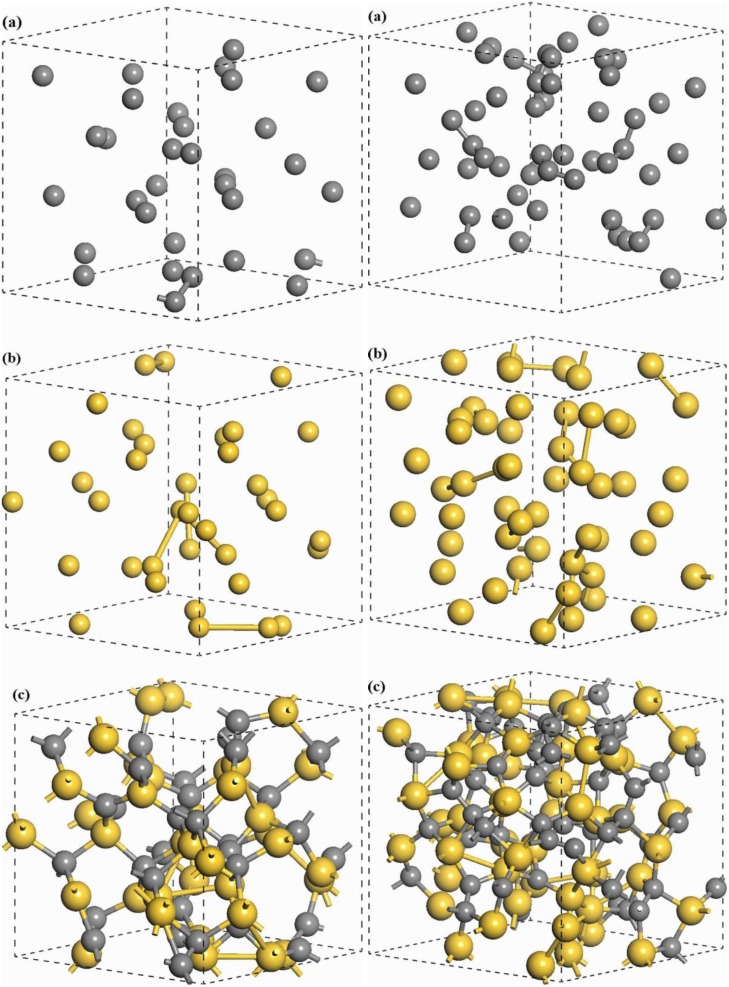
Structures of the amorphous silicon carbide generated by Fast (left, 64 atoms) and DMol3 (right, 108 atoms). (**a**) Carbon structure; (**b**) Silicon structure; (**c**) Silicon carbide structure.

The simulational results for the atomic structures are presented in Figure 21; the left column refers to a supercell of 64 atoms, 32 carbons and 32 silicons, amorphized with Fast, and there are short chain-like structures of carbon. The right column corresponds to an amorphous structure with a total of 108 atoms, 54 carbons and 54 silicons, amorphized with DMol3. The carbon structure also shows short chain-like configurations, suggesting that there cannot be a complete chemical order.

The maximum bond length in Figure 21 was determined as the distance at which the first minimum occurs in the corresponding pRDF in the graphs of Figure 3 of Reference 86, as follows. For the 64-atom sample, generated with Fast, we have the maximum length of the carbon-carbon bonding as C–C: 1.55 Å (tetrahedral carbon); the maximum bond length for silicon-silicon is Si–Si: 2.55 Å and the silicon-carbon maximum bond length is Si–C: 2.25 Å. For the 108-atom sample, generated by DMol3, we have the same values for the maximum lengths of the interatomic bonding: the carbon-carbon bonding, C–C, is 1.55 Å; the Si–Si is 2.55 Å and the Si–C is 2.25 Å.

Even though the maximum lengths of the bonds are the same for both structures the chemical ordering may be different. The chemical order can be estimated with a formula used by Tersoff [88]:

χ = *n_A–A_*/*n_A–B_*

where *n_A–A_* is the number of homonuclear bonds and *n_A–B_* is the number of heteronuclear bonds. Therefore when χ = 0 complete chemical order is implied whereas χ = 1 implies complete chemical disorder. For the 108-atom sample χ = 0.05, which implies an almost complete chemical order; for the 64-atom sample χ = 0.065, similar to the larger supercell.

**Figure 22:**
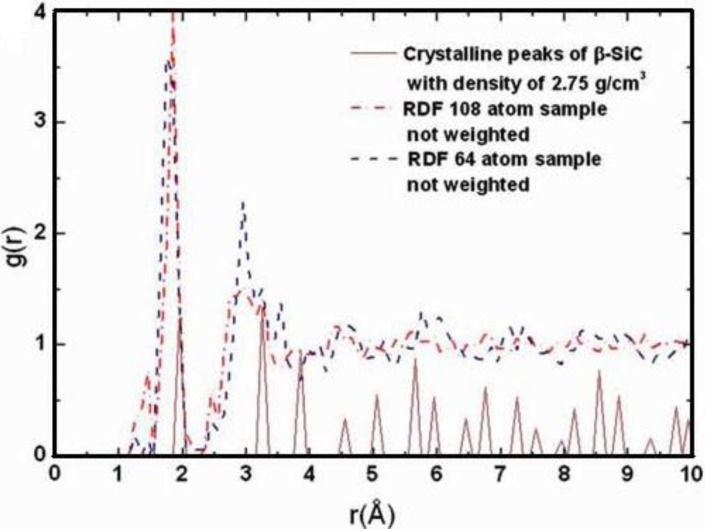
Comparison of the two simulated total RDFs with the crystalline structure of β-SiC. The second crystalline peak seems to appear in the amorphous RDFs.

In Figure 22 we present a comparison of the two simulational, total, bare, RDFs with the crystalline structure of β-SiC. The second crystalline peak seems to appear in the amorphous structure of the RDFs, but it may be a coincidence since no other crystalline peak agrees with sharp structures of the amorphous material.

**Figure 23:**
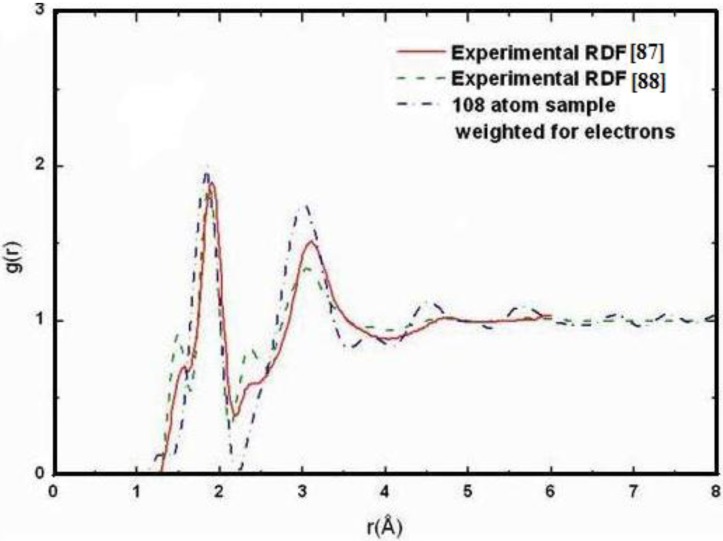
Comparison of the total RDF for the 108-atom sample, weighted for electrons, with the two experimental results [89,90].

Figure 23 is a comparison of two experimental results [89,90] with the total RDF for the 108-atom sample, weighted for electrons. The agreement is good since the positions of the peaks coincide and so do the positions of the shoulders. One point of discrepancy is the fact that for the simulated RDF there is a ‘zero’ minimum between the first and the second peaks, feature that does not appear in the experimental results. This discrepancy may be due to the way the amorphous samples are prepared experimentally, since they are disordered by irradiating a crystalline sample.

#### 4.4.3. Summary

The amorphous binary alloys of group IV elements, like SiC and SiGe, are extremely interesting since there are chemical characteristics that are notably different, as in Si and C, and very similar, as in Si and Ge. This is one of the reasons for our interest in these alloys. The other reason is to study the chemical ordering in these two systems since C and Si are very different and Si and Ge are very similar in their atomic electronic structures. More extensive results are reported in [85].

### 4.5. Amorphous Chalcogenide Alloys: The Case of a-GeSe_2_ [91,92]

Chalcogenides are materials that have proven to be technologically very useful, and their amorphous phases have been studied systematically. The role that selenium played in the development of xerography is well known. When alloyed with germanium and indium and made into an amorphous phase it displays interesting properties. For example the character of the interatomic forces in an alloy of Ge–Se can be modified by altering the composition. As Salmon comments, just as pure Ge undergoes a semiconductor to metal transition on melting, the nature of the interatomic bonding changes from metallic to covalent by varying the selenium concentration [93]. There are some other properties that are modified upon alloying germanium with selenium (see [93] and references contained therein).

#### 4.5.1. Preamble

The Ge-Se system has a large glass forming region that extends over the germanium concentration from 0.0 to 0.43 [94]. The amorphous phases are covalently bonded and the connectivity and properties of these networks can be altered by modifying the Ge–Se ratio. In this work, we only report the amorphous GeSe_2_ alloy.

There are a few a*b initio* CPMD studies on amorphous GeSe_2_ (See Section 2). Recently Massobrio and Pasquarello generated amorphous networks by cooling a liquid 120-atom supercell [22,23]. They used 40 Ge and 80 Se at constant temperature. They also used a cubic periodic supercell of size 15.16 Å with density 4.38 g/cm^3^. There is another *ab initio* work based on the local density-functional method of Sankey and coworkers [24,25,26]. Cappelletti *et al.* [25] studied vibrations in glassy GeSe_2_ with a 63-atom supercell. They used the *melt-and-quench* process on their model. There were 20 Ge and 43 Se in this model placed randomly on sites of a diamond lattice; after the thermal process they removed one Se that was connected with other two Se forming a trimer. They then again applied a thermal process and put a Ge-atom in to get the exact stoichiometry for GeSe_2_. The resulting density was 4.20 g/cm^3^. They compared their vibrational results with experiment. In their second work on *a-*GeSe_2_, Cobb *et al.* reported a 216-atom supercell, with a density close to experiment [26]. As mentioned, in this *ab initio* study they obtained topological, vibrational and electronic properties.

#### 4.5.2. Results and Analysis

In order to study amorphous GeSe_2_ we applied our computational simulation protocol and in this case we used the DMol3 code. We obtained an *ab initio* generated amorphous network with a concentration of 66% selenium and 33% germanium. We started with a cubic crystalline supercell of edge length 16.66 Å with 50 atoms of germanium and 100 atoms of selenium. In the supercell all Ge atoms were connected to four Se atoms and no Ge–Ge bonding was allowed. The supercell was linearly heated using DMol3 from 300 K to 970 K, a value below the melting temperature, in 100 steps of 15 fs and immediately cooled down to 0 K in 145 steps. The heating/cooling rate was 6.70 × 10^15^ K/s. The atoms were allowed to move within the cell with periodic boundary conditions, and its volume was determined by the corresponding experimental density and concentration. We next subjected it to an annealing process at 300 K, with a quenching step down to 0 K, a variant of the *undermelt-quench* approach. At the end a geometry optimization was carried out to find the amorphous structure at the local energy minimum. The density considered was 4.13 g/cm^3^, 3 % less that the crystalline one [26]. The amorphization process was performed with a pseudopotencial *dspp* for the electron treatment, and for the geometry optimization an *all electron* approach was used. We used double numerical orbitals with polarization basis set, two atomic orbitals for each occupied state and functions with angular momentum higher than that of the highest occupied state in the free atom; the cutoff radius used was 5 Å both for the amorphization and for the optimization.

**Figure 24:**
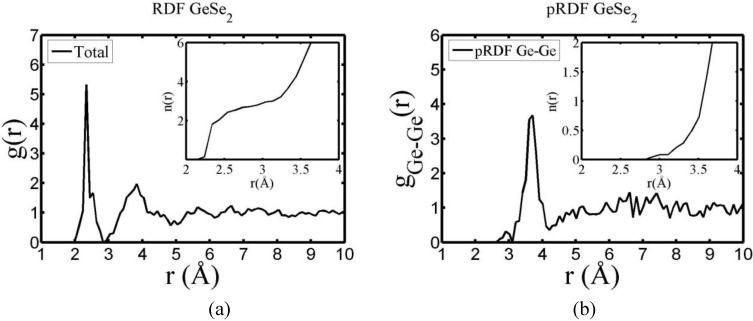
(**a**) Total RDF for the system a-GeSe_2_; (**b**) Partial Ge–Ge RDF for the same system. Insets show the corresponding average coordination numbers.

**Figure 25:**
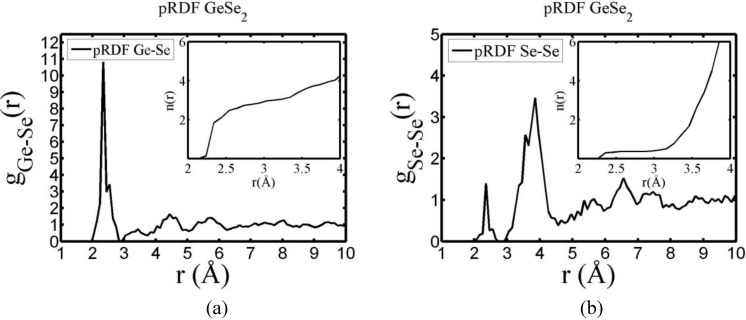
(**a**) Partial Ge–Se RDF for the system a-GeSe_2_; (**b**) Partial Se–Se RDF for the same system. Insets show the corresponding average coordination numbers.

As before, we used the minimum of the RDF obtained in this work, Figure 24 and Figure 25, to determine the maximum bond length. We considered that two atoms were connected if the distance between them was less than or equal to 2.84 Å, the first minimum in Figure 24(a). With this bond length the average coordination number is 2.71, which is in good agreement with experiment 2.67 [95]. In our resulting amorphous supercell there was no homopolar Ge–Ge bond. This fact can be seen in the Ge–Ge pRDF (Figure 24(b) and Table 5) which shows no atoms before 2.80 Å. In Table 5 the nearest neighbor peaks from the pRDFs obtained in our simulations are given. The short-range order for our model predicts that 68% of the Ge atoms form Ge(Se_1/2_)_4_ tetrahedra, 30% are three-fold coordinated and 2% are one-fold coordinated. No five-fold coordinated Ge and no ethane-like structures were found. The bonding defects for selenium are as follows: 1% of four-fold coordinated atoms; 21 % with three-fold coordination and 5% with one-fold coordination. We also calculated the tetrahedral distribution for our structure and found that 94% were corner-sharing and only 6% were edge-sharing.

**Table 5:** Nearest neighbor peaks from the pRDFs obtained in our simulations compared to experiment, Salmon and Petri [95], and to other *ab initio* work [22,26].

Partial	This work	Massobrio *et al.*	Cobb *et al.*	Salmon and Petri
g_GeGe_	2.91 Å	2.50 Å	2.46 Å	2.42 Å
g_GeSe_	2.34 Å	2.37 Å	2.37 Å	2.36 Å
g_SeSe_	2.36 Å	2.37 Å	2.40 Å	2.37 Å

#### 4.5.3. Summary

We have simulated the amorphous alloy GeSe_2_ by using a variant of the *undermelt-quench* approach. The results show good agreement with the topological properties obtained from other *ab initio* works and from experiment. We conclude that there are no Ge–Ge nearest neighbors (with homopolar bonds). We found that 68% of the germaniums formed Ge(Se_1/2_)_4_ tetrahedra; 94% were corner-sharing and 6% were edge-sharing tetrahedra. For completeness we mentioned that we have also simulated *a*-InSe alloys for several concentrations (over 20) to be able to calculate their topological properties, their eDOS, the corresponding vDOS and their optical gaps to contribute to the engineering of the gap, but the results are the subject of work in progress [91].

### 4.6. Aluminum-based Amorphous Alloys: AlN [96] and Al12at%Si [97,98,99,100]

Liquid and amorphous metallic systems have proven difficult to model. Some efforts have relied on the use of parameterized classical potentials of the Lennard-Jones type or geometric hard sphere simulations, but first principles methods have been rarely used. With the experimental development of many and more complicated BMGs a potential dilemma appears. It is necessary to calculate larger samples of materials that better resemble the bulk properties, but it is also necessary to do it as reliably as possible. This implies that in our *ab initio* approach we need to be able to calculate larger supercells of bulk metallic glasses to, in principle, make the results relevant. Of course the computational demand is enormous but perhaps some of the properties of these metallic systems can be representatively obtained with tractable supercells, and this is what we are studying. For that reason we have applied our methods to pure amorphous aluminum [101], *a-*AlN and *a-*AlSi alloys. Here we report only some results for the aluminum silicon system. For the aluminum nitrogen system we refer the reader to Reference 96. In Section 4.7 we analyze the applicability of our approach to amorphous CuZr; similarities with the corresponding BMG appear.

#### 4.6.1. Preamble

Metallic glasses and amorphous metallic alloys are presently in the forefront of metallurgical research. Their technological applications make it necessary to understand them as closely as possible, and for this, computer simulations are in order. Some computer simulation efforts have relied on the so-called embedded atom method [102,103] or tight binding methods [104] to overcome the existing limitations of using pair potentials for describing the metallic bond. Some efforts have relied on the use of parameterized classical potentials of the Lennard-Jones type or geometric hard spheres. Some of the first principles methods used in the field are due to work developed by our group [96,97,98,99,100,101].

We have studied the structural properties of amorphous aluminum [101] and its alloys [96,97,98,99,100] with LHMD; the results obtained are promising to describe some of the properties of these materials. The aluminum-silicon system is an important technological alloy. From the point of view of its phase diagram, this system is relatively simple; it is a eutectic at 12 atomic % Si with no ordered phases. There is some solubility of Si in fcc Al, but almost no solubility of Al in diamond-structured Si [105]. The solidification process is much different, due to the formation of faceted interfaces between crystalline Si and the liquid Al–Si. This leads to very interesting solidification microstructures [106]. Small additions of a third element can also affect the microstructure [107], but are not discussed here.

To perform LHMD we used the same computational tools applied to previous systems. The Harris functional is used and therefore no selfconsistency is employed. The core is taken as full since all electrons are considered, and for the amorphizing/liquefying process a double-numeric standard basis set was chosen with a cutoff radius of 5 Å. The physical default time step is 2.4 fs but a time step approximately 4 times the default was finally utilized: 9 fs. To study Al12at%Si we built a supercell with 125 atoms from simple cubic structures and randomly changed 15 aluminums for silicons to generate the eutectic concentration. The volume was adjusted to (12.8379 Å)^3^ to obtain the crystalline density 2.66 g/cm^3^. To generate the amorphous supercell, *a*-Al12at%Si, we applied the *undermelt-quench* approach with a variant: starting at room temperature the sample was heated in 100 computational steps to just *below* the melting temperature. The sample was maintained at this temperature during 100 steps (plateau); afterwards it was cooled down to 0 K at the same thermal rate. The structure was optimized to find the final atomic topology in local equilibrium. To generate the liquid-amorphous sample [96], *la*-Al12at% Si, we heated the supercell to just *above* the melting temperature, maintained it at this temperature during 100 steps (plateau), and then cooled it down to 0 K at the same thermal rate.

#### 4.6.2. Results and Analysis

**Figure 26:**
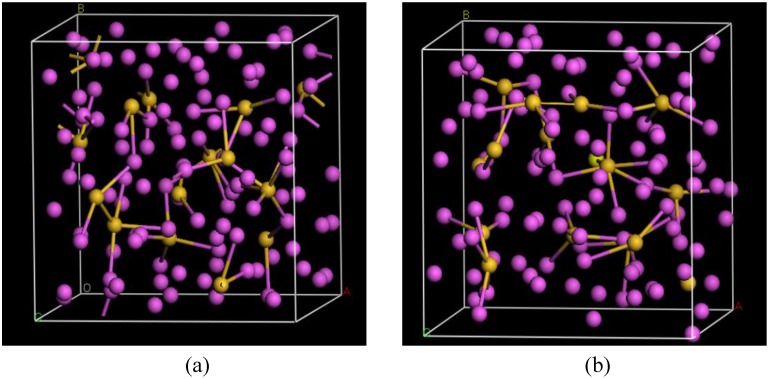
(**a**) Amorphous atomic structure of the 12 at %Si supercell, *a*-Al12at%Si. The plateau temperature is 840 K, just below the melt; (**b**) Liquid-amorphous (see Refenrence 96) atomic structure of the 12 at %Si supercell, *la*-Al12at%Si. The plateau temperature is 920 K, above the melt. Silicon atoms are in yellow.

Figure 26 are the atomic topologies found (a) for the amorphous sample and (b) for the liquid-amorphous sample (see Reference 96). At first sight the two structures seem different but it is necessary to quantify the features of each one, and this is accomplished by calculating and analyzing the RDFs, and that is why we obtained total and partial RDFs for the two supercells. Nevertheless it is worth noting that the silicons in the amorphous structure (Figure 26(a)) seem to be more clustered than the corresponding ones in the liquid-amorphous structure (Figure 26(b)).

**Figure 27:**
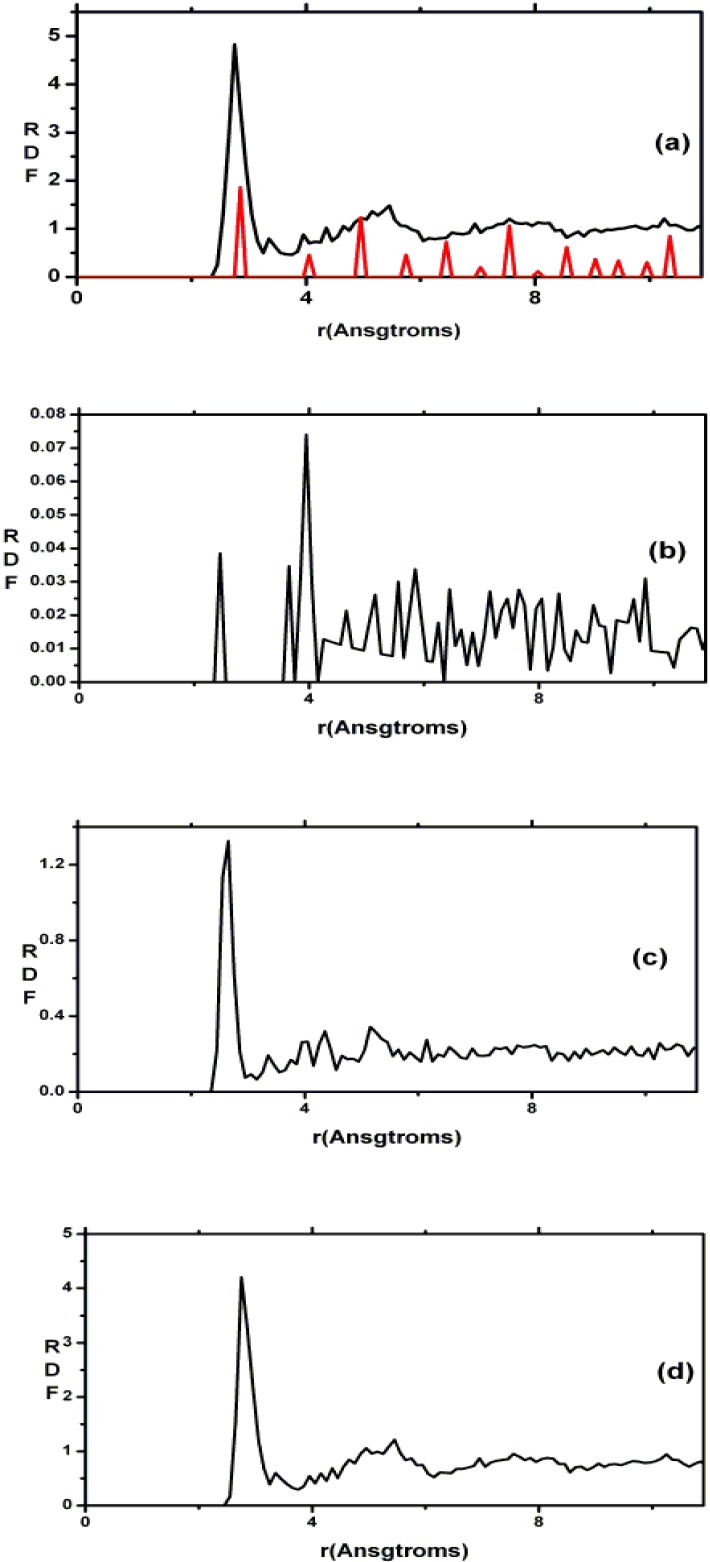
The system *a*-Al12at%Si relaxed. (**a**) Total RDF; the pRDFs are (**b**) Si–Si; (**c**) Al–Si and (**d**) Al–Al for the 15 Si and 110 Al cell. The red peaks in (**a**) are the atomic positions of the fcc structure.

**Figure 28:**
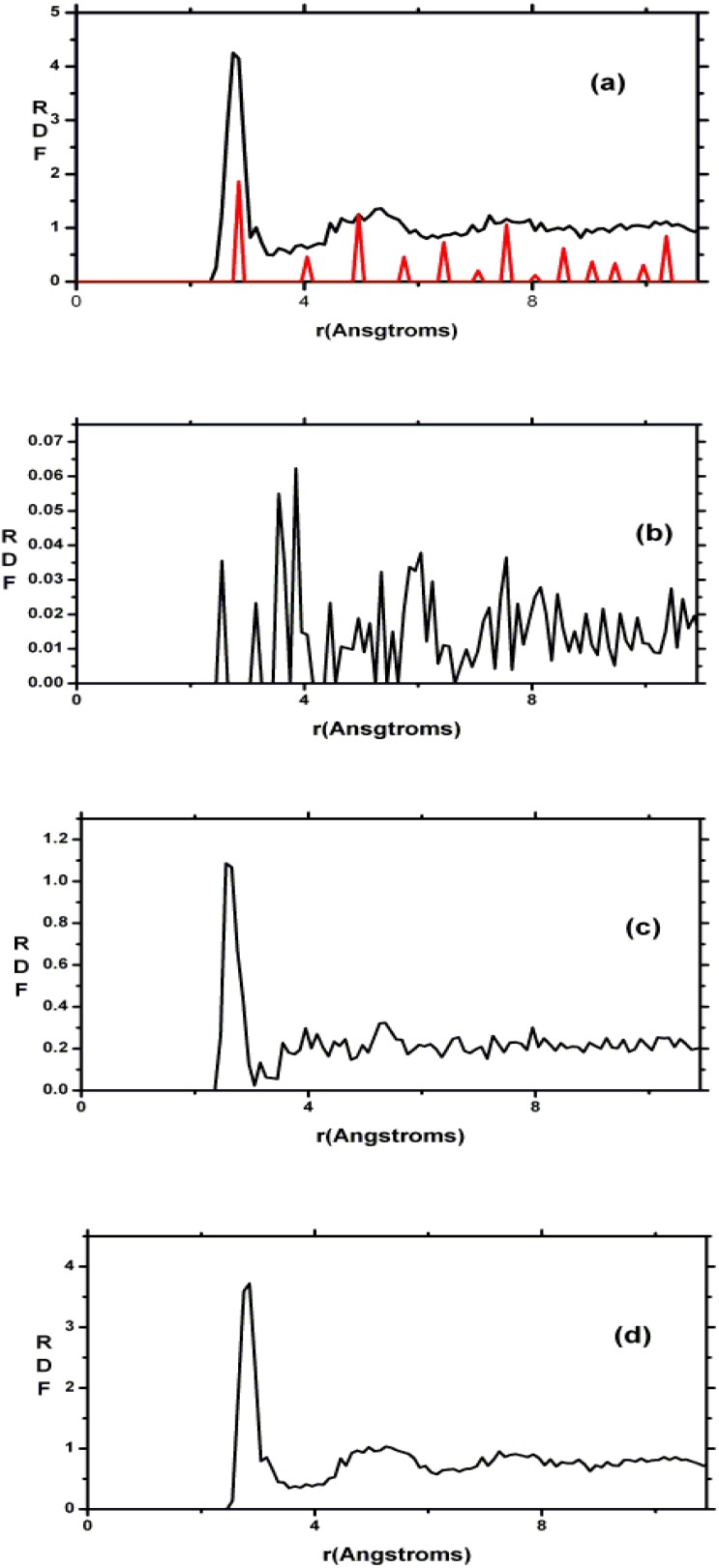
The system *la*-Al12at%Si relaxed. (**a**) Total RDF; the pRDFs are (**b**) Si–Si; (**c**) Al–Si and (**d**) Al–Al for the 15 Si and 110 Al. The red peaks in (**a**) are the atomic positions of the fcc structure.

Figure 27 shows the total and partial RDFs for the amorphous sample (Figure 26(a)). It is clear that the first prominent peak of the total RDF, Figure 27(a), corresponds to the Al–Al bonds (2.75–2.85 Å) and this can be corroborated by looking at the partial RDF for Al–Al, Figure 27(d). The Si-Si partial, Figure 27(b), indicates that, as mentioned above, there seems to be some degree of clustering of the silicon atoms since three highly localized peaks appear in the corresponding partial RDF (2.45, 3.65 and 3.95 Å). The total RDF (Figure 27(a)) reflects the bimodal structure of the Al–Al partial RDF (Figure 27(d)). For the Al-Si partial RDF (Figure 27(c)) there is a prominent peak at 2.55–2.65 Å. Figure 28 shows the total and partial RDFs for the liquid-amorphous structure of Figure 26(b). Again, the first prominent peak of the total RDF, Figure 28(a), corresponds to the Al–Al bonds (2.75–2.85 Å) and this can be corroborated by looking at the partial RDF for Al–Al, Figure 28(d). The Si–Si partial, Figure 28(b), is more evenly distributed as a function of *r* as can be seen from the position of the first four peaks (2.55, 3.15, 3.55 and 3.85 Å). The total RDF (Figure 28(a)) shows a bimodal structure but the Al–Al partial RDF (Figure 28(d)) does not. As in the amorphous case the Al–Si partial RDF (Figure 28(c)) presents a prominent peak at 2.55–2.65 Å.

Experimentally some RDFs have been determined for amorphous Al–Si and Al–Si–Sb alloys [107], but the authors report only the position of the Si–Si second peak, 3.7 Å, which should be compared to 3.65 Å for our amorphous sample and to 3.15 Å for our liquid-amorphous sample.

**Figure 29:**
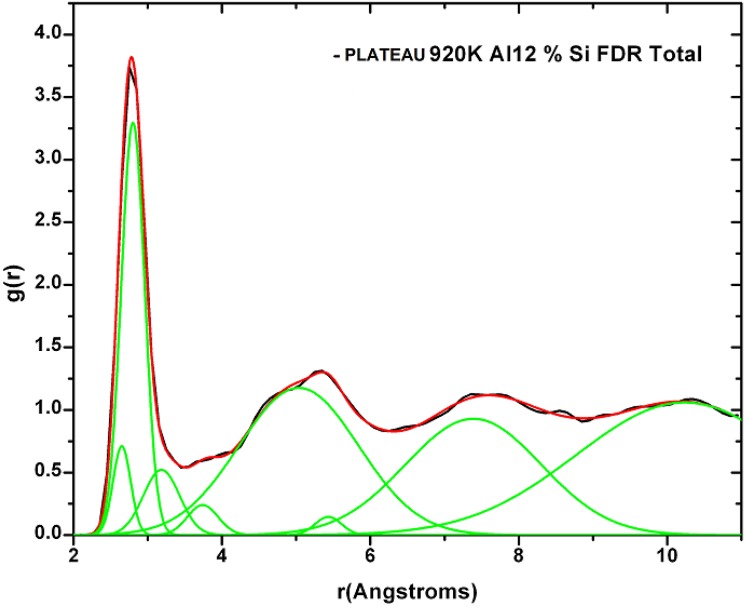
Deconvolution of the total RDF (black curve) for the *la*-Al12at%Si eutectic alloy. We obtain the smallest number of Gaussians (green curves) whose sum (red curve) reproduces the corresponding RDF. For the first neighbors we sum the areas that contribute to the first peak of the pertinent RDF.

As mentioned before a difficult problem in amorphous materials is finding a criterion to determine when two atoms are considered bonded. However, in this work we use a novel deconvolution method reported in Reference [99] to carry out the estimation of the coordination numbers. Using this method, illustrated in Figure 29, the coordination number for the amorphous eutectic alloy is 11.48, whereas each aluminum atom is surrounded by 11.96 atoms on the average and each silicon atom is surrounded by 7.96 atoms on the average. This suggests that the aluminum structure in the short range is that of an icosahedral cluster and that the silicon short range structure is closer to a metal. The coordination number for the liquid-amorphous eutectic alloy is 10.56, whereas each aluminum atom is surrounded by 10.92 atoms on the average and each silicon atom is surrounded by 7.92 atoms on the average. This suggests to us that the aluminum structure in the short range is, as expected, that of a liquid. More studies are under way to fully analyze the consequences of this deconvolution method and its relevance for calculating coordination numbers.

#### 4.6.3. Summary

The differences between the *amorphous* and the *liquid-amorphous* structures are notable and the distribution of silicon is more uniform in the liquid-amorphous sample than in the amorphous sample. A bimodal behavior is present in the total RDFs. This bimodal feature has appeared in metallic liquids results [108] and in some geometrical results [109] and is characteristic of metallic systems [110]; this feature has been associated to the appearance of dominant icosahedral clusters [28].

Generating simulated amorphous structures of metallic materials is a difficult task, especially when there are not enough experiments to compare with and to guide the selection of the thermal process. Since this is the case, our results may be considered predictive and indicative of possible structures based on which properties can be calculated. The deconvolution method gives us more information about the atomic average position and the atomic environment around each atom; this leads to coordination numbers that may be more adequate to describe amorphous and liquid materials.

### 4.7. Amorphous Copper Zirconium Alloys. A Simple BMG-like Material: Cu_64_ Zr_36_ [111]

Bulk Metallic Glasses have become fashionable because of their possible applications in industry. These metallic materials obtained from the melt have many unique applications from structural components to microcomponents [3]. Their properties, like good hardness and high corrosion resistance, are highly desirable since they may be used as pressure sensors, gears for micromotors, magnetic cores, *etc.* Producing glassy metals is a very difficult experimental endeavor since the atomic constituents are highly mobile and unless the cooling rates are superior to 10^6^ K/s the systems in general crystallize. This fact usually restricts the size and stability of the products. Recently however several laboratories in the world have made important progress and BMGs have been produced by increasing the number of components that are incorporated into the matrix, thus needing lower cooling rates. For example, some 17 years ago Peker and Johnson developed a pentary metallic glass with a cooling rate of 1 K/s: Zr_41.2_Ti_13.8_Cu_12.5_Ni_10.0_Be_22.5_ [112]. In the last 40 years BMGs have increased considerably in number and in size and it is assessed that, at present, more than a thousand different bulk metallic glasses are known, some of them in sizes of several kilograms [3]. In this section we analyze the applicability of our approach to amorphous CuZr and similarities with the corresponding BMG will appear.

#### 4.7.1. Preamble

During the 1980s much progress was made in the experimental study of amorphous metallic multicomponent alloys—ternary, quaternary, *etc*. However, the main goal was the development of alloys whose properties could be studied due to the simplicity of their composition, *i.e.*, binary alloys. A simple copper–based alloy which has gained relevance in the last decade is the CuZr system because it is relatively easy to produce in its glassy phase [113].

Some experimental work on the topology of the *g-*Cu_64_Zr_36_ alloy has been reported (Section 2). Wang and co-workers performed an analysis of the structure in *g-*Cu_64.5_Zr_35.5_ by means of XRD [29], finding an icosahedron-like clustering preference among other short range order polyhedra. Mattern *et al*. performed an experimental study which involved XRD and neutron scattering for three different compositions: Cu_100−*x*_Zr*_x_* (*x* = 35, 50, 65) [30]. They found an average coordination number of 13.2, but they did not assert any particular short-range order structure present in their samples.

As mentioned in Section 2, Wang *et al*. also performed AIMD and RMC studies on a sample with the same concentration they used for the XRD analysis [29]. By comparing AIMD with XRD results, and RMC with EXAFS, they obtained the 3D structures of the samples so the short range ordering could be established. Likewise, Mattern *et al*. carried out an RMC study [30]. They used the simulational part to resolve the pRDFs and consequently to obtain their coordination number. In addition to this, Jakse and Pasturel reported an AIMD study of the *g-*Cu_64_Zr_36_ alloy [31,32]. They obtained a coordination number closer to the one found by Mattern and co-workers, *i.e.*, 13.1.

It is noteworthy that these computational works have in common the use of plane waves as basis sets and a thermal procedure which leads to a metallic glass obtained by cooling from the melt. Therefore we decided to use a variant of the *undermelt-quench* protocol that excludes the stress relieving cycles since for this system they are very time consuming in the computational procedure; we also used the quantum mechanical tools mentioned before to generate the structure of an amorphous alloy to compare with the corresponding metallic glass. The working code was DMol3 and since the Harris functional cannot handle d-band elements appropriately we decided to use a self-consistent approach and pseudopotentials. Also, since we have not found any previous work reporting the application of linear combination of atomic orbitals (LCAO) as basis sets for this alloy, we decided to undertake an LCAO study to find out the possible influence of our approach on the final structures. The LDA and the exchange-correlation functional due to Perdew and Wang with corrections by Ceperley (PWC) were used [114]. The calculations were spin-restricted with double numerical basis sets, which include d polarization functions (dnd). Given that *ab initio* calculations need an intensive computational effort, we used two different pseudopotentials (*pp*): (a) *dspp* (DFT semilocal pseudopotential) which replaces the core electrons with a simple potential, thus speeding the process, and (b) *vpsr*, which takes into account core and valence electrons performing scalar relativistic corrections on them, a more demanding pseudopotential. In order to obtain better dynamical results, and since we have a system made up of heavy elements, we used two different time steps based on the default time step (DTS) value of 3.57 fs: 10.71 fs (3DTS) and 14.28 fs (4DTS), so that the system has enough time to evolve. We used periodic boundary conditions and a cut-off distance of 4.4 Å for Cu and 5.3 Å for Zr.

Starting with an fcc supercell of 108 atoms of copper, 39 atoms were randomly substituted by Zr atoms, reaching a density value of 8.06 g/cm^3^. From room temperature, we linearly heated the sample in 100 steps to 1,223 K, 10 K below the melting point [115]. Afterwards the samples were quenched to 0 K in 133 steps, the *undermelt-quench* approach. Thereupon we had two heating (cooling) rates: 0.86 × 10^15^ K/s for 3DTS and 0.65 × 10^15^ K/s for 4DTS. At the end of this thermal process some stresses emerged within the samples, hence, we geometry optimized them to relax the structures so that they could reach a local energy minimum.

#### 4.7.2. Results and Analysis

**Figure 30:**
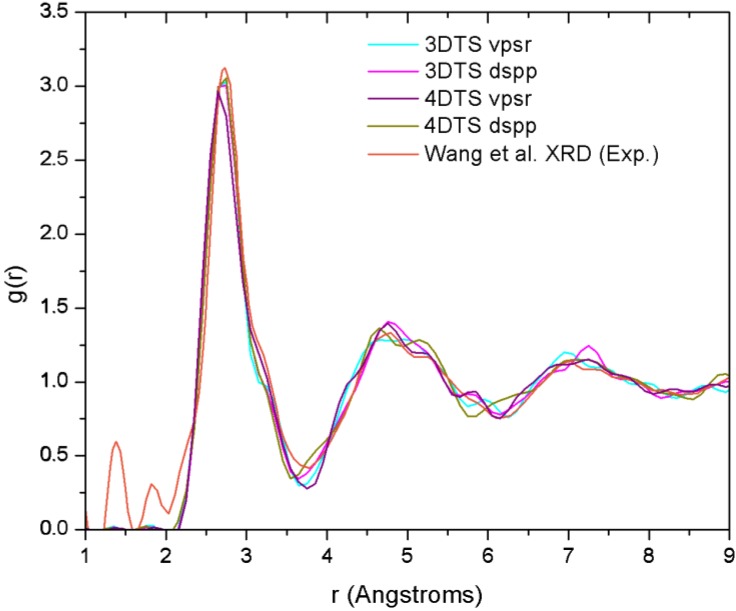
Comparison of our RDFs and the experimental glassy RDF of Wang *et al*. [29]. Our results were weighted for X-ray scattering in order to compare them with experiment.

**Figure 31:**
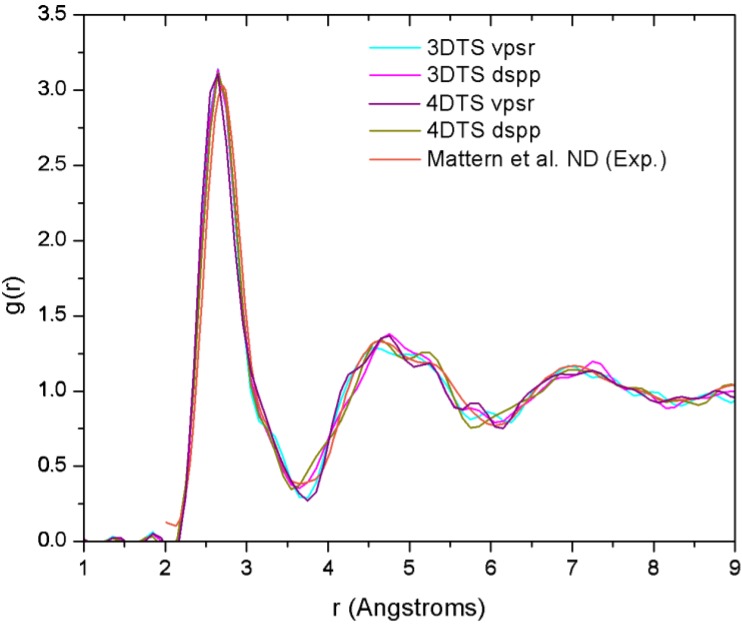
Comparison of our RDFs and the experimental glassy RDF of Mattern *et al*. [30]. Our results were weighted for neutron scattering in order to compare them with experiment.

**Figure 32:**
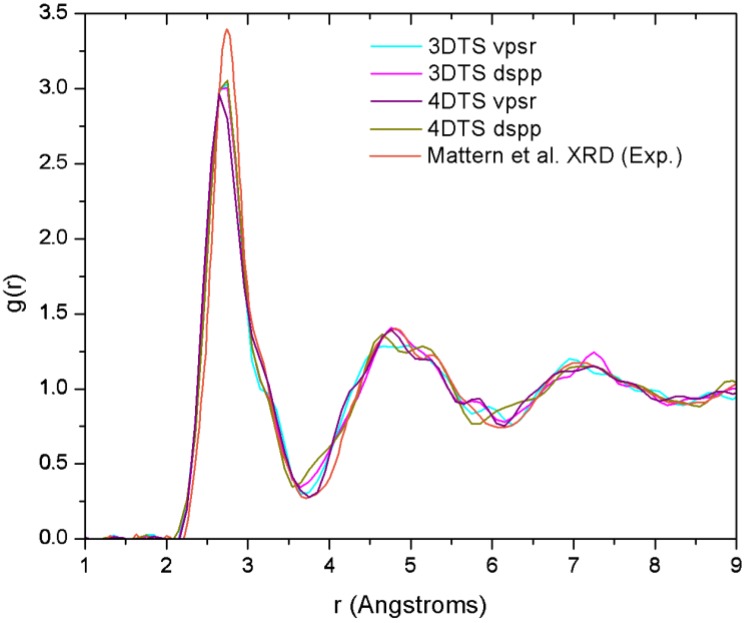
Comparison of our RDFs and the experimental RDF of Mattern *et al*. Our results were weighted for X-ray scattering in order to compare them with experiment.

We obtained the radial distribution functions (RDFs) of the four samples (3DTS vpsr, 3DTS dspp, 4DTS vpsr and 4DTS dspp), weighted them correspondingly and compared them with the experimental curves for the glassy alloy reported by Wang *et al*. [29] and Mattern *et al*. [30]. In Figure 30, Figure 31, and Figure 32 we see a remarkable resemblance with the experimental RDFs (two for X-rays and one for neutrons). Our results reproduced the shape of the first peak very well, and the presence of the right shoulder. However we observe that there are slight differences between our RDFs and the experimental glassy ones regarding the position of the first peak, the first valley and the shape of the second peak. Our RDFs exhibit the so-called splitting of the second peak or bimodal second peak, which is a feature present in amorphous and liquid metallic alloys as mentioned before.

From Table 6 we can see that there are some discrepancies in the partial coordination numbers with respect to the experimental values. We computed the coordination numbers by integrating 4*π* *r^2^* *ρ*_0_ *g*(*r*) up to the first minimum present in each RDF. Here *ρ*_0_ is the number density and *g*(*r*) is the RDF. We have undercoordination in the Cu–Cu and Zr–Zr partial numbers, and an overcoordination in Cu–Zr and Zr–Cu with respect to the glassy results. Notwithstanding, our values do not show a significant difference among them. In addition, it is also interesting to notice that our total coordination number agree well with the value reported by Mattern *et al*. We think that these differences may arise from the fact that we never melted the alloy in order to obtain the amorphous metal, *i.e*., we are not simulating a metallic glass but an amorphous metallic system. However, it is of paramount importance to mention that our results clearly expose a resemblance between the non-glassy amorphous alloy and the metallic glass, that is, they have topological properties that are very similar.

**Table 6:** Comparison of our total and partial coordination numbers with the experimental values of Mattern *et al*. [30].

	*N*_Cu–Cu_	*N*_Cu–Zr_	*N*_Zr–Zr_	*N*_Zr–Cu_	*N*_T_
Mattern *et al.^a^*	6.7	3.9	5.9	7.6	13.2
*3DTS vpsr^b^*	4.3	5.3	2.3	9.4	14.0
*3DTS dspp^b^*	4.1	5.4	2.0	9.6	13.6
*4DTS vpsr^b^*	3.9	5.4	2.2	9.5	13.7
4DTS *dspp^b^*	3.7	5.1	2.0	9.1	13.4

*^a^* Refrence [30] (The partials ***N*_ij_** were obtained via Reverse Monte Carlo simulations); *^b ^* This work (Our ***N*_T_** results were weighted for XRD).

As for the use of different *pp*s, we can say that there are no big disparities between the *dspp* approximation and the *vpsr* results. Regarding the energies of our samples, we noticed that both the *dspp* and *vpsr* runs for a time step 3DTS exhibit an energy minimum which does not exist in the 4DTS runs. Therefore, we consider the time step 3DTS (10.71 fs) as the optimal time for the amorphization procedure; also, since the *dspp pp* leads to reliable results without compromising the quality, the adequate parameters for this alloy are: *dspp pp* and 3DTS.

#### 4.7.3. Summary

Theoretical studies via simulational modeling of amorphous metals have been shown to be in agreement with the topological properties of the experimental data. By applying a variant of the *undermelt-quench* approach we obtained amorphous samples which resemble exceedingly well the experimental results for the metallic glass, not only qualitatively, but also quantitatively. From this work we come to the following conclusions:

(a) The disparities in the partial coordination numbers with respect to those experimentally reported, are related to the thermal process chosen, *i.e.*, we always stayed under the melting point and therefore the metallic glass was never obtained. Moreover, from the AIMD studies and the reported experimental values, only Jakse and Pasturel [32] establish with precision the method they used to compute the coordination numbers.
(b) The energies of the samples let us know that a time step of 10.71 fs, along with the *vpsr pp*, lead to a more metastable amorphous structure. Therefore the use of the *dspp pp* is indicated.
(c) The amorphous alloy shares structural characteristics with the metallic glass, namely, the RDFs are very similar and the total coordination values are also very similar. Nevertheless differences are to be noted.

These points suggest that our approach for the generation of amorphous metals is on the right track. However, we must fine-tune the process in order to handle a larger number of atoms, to calculate electronic and vibrational properties of such materials, and to deal with BMG.

## 5. Amorphous Alloys: Their Properties [1]

The agreement with experiment of the topological properties of the amorphous structures that we have generated using our simulational protocol: *undermelt-quench* + Fast, or some of its variants, gives us some degree of confidence in our method. Nevertheless, agreement between experiment and atomic topology through RDFs, measured and simulated, is only one aspect of the problem since it is well known that a given RDF can represent many atomic structures. Initially we have no way of knowing if the specific atomic structure that we have generated has the correct physical properties. For this reason it is necessary to calculate some physical properties of these structures and to compare them with experiment. We therefore report the calculations of some physical properties: the eDOS and the electron energy gaps of *a*-SiH, the eDOS and the optical gaps of *a*-SiN and because of its relevance for the equilibrium properties of the generated supercells, we report results of a calculation of the vDOS for pure *a*-Si that is presently been applied to some of the materials mentioned above. For the calculation of these properties all the amorphous atomic structures used were generated by means of our simulational protocol, and the agreement with experiment is very good.

### 5.1. Electronic Energy Gaps of Amorphous Hydrogenated Silicon [48]

As mentioned in Section 4.1 two structures of hydrogenated silicon were generated: one identified as the sample 2.44/0.46, the other as 10/2 because of the time steps used in the simulations. Once these atomic structures were constructed and their respective RDFs obtained (See Section 4.1) we analyzed their eDOS at the Γ-point of the artificial periodic Brillouin Zone, a widespread practice in the field. We carried out energy calculations using both Fast and the full Kohn-Sham DFT approach implemented in the *ab initio* commercial code DMol3 to obtain the energy levels and eDOS curves of the final amorphous atomic structures, using LDA. In DMol3 a double numerical basis set that includes *d*-polarization of the atoms (DNP) and the frozen-inner-core orbital approximation along with a medium grid for the calculation of integrals were used. The SCF density parameter that specifies the degree of convergence for the LDA density was set at 10^−6^. We then obtained eDOS curves by broadening the discrete eigenvalues spectra generated with Fast and DMol3 for the final amorphous structures.

**Figure 33:**
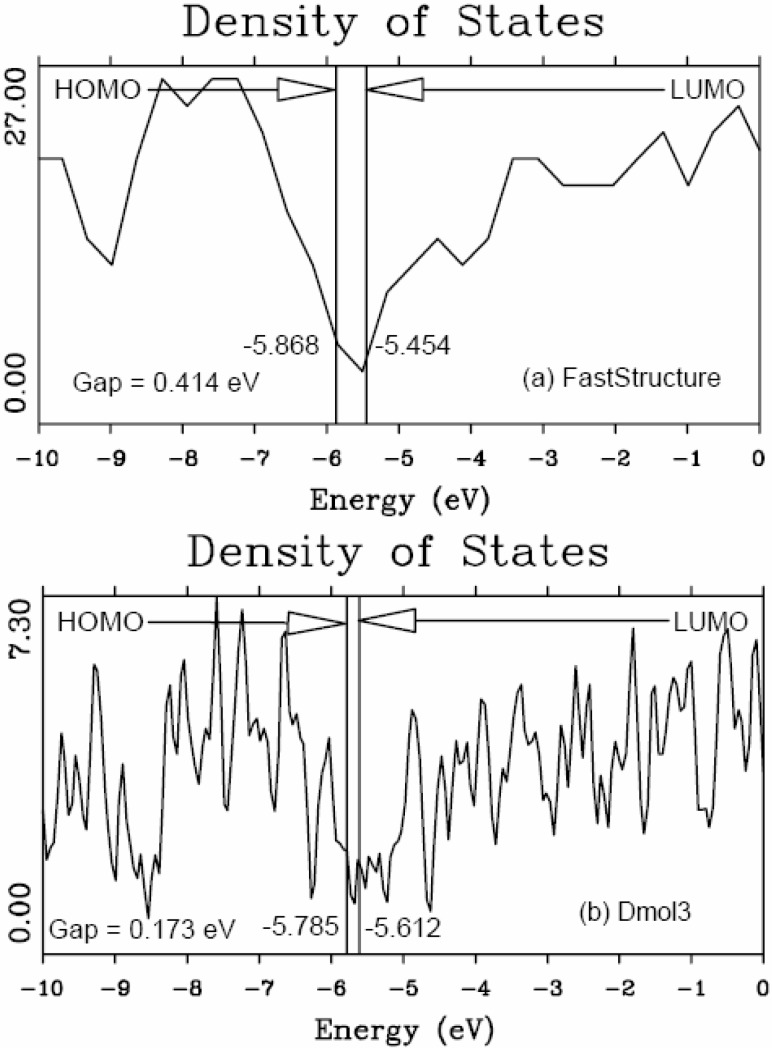
eDOS curves for the 2.44 fs cell of *a-*Si calculated using Fast, curve (**a**), and DMol3, curve (**b**). HOMOs, LUMOs and gaps are indicated.

**Figure 34:**
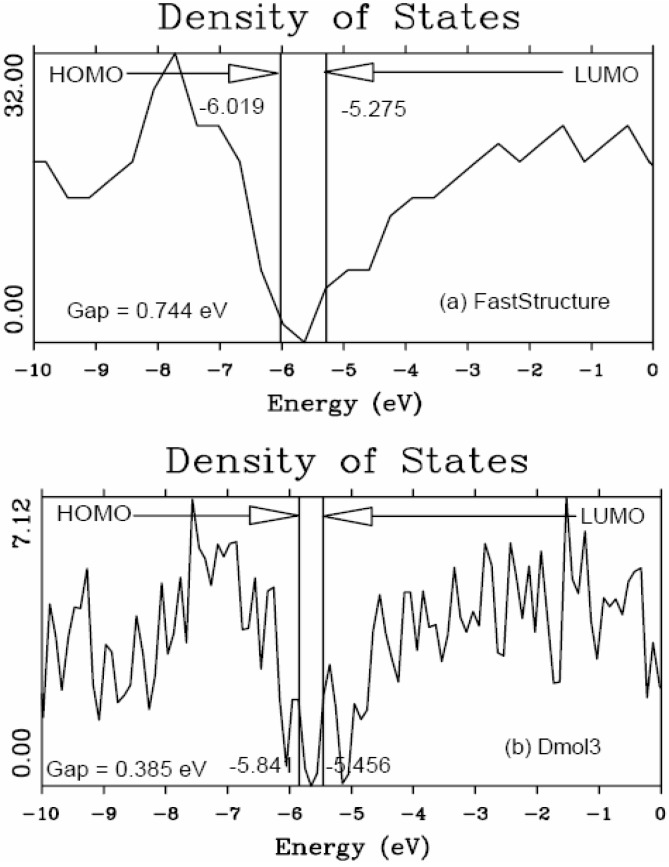
eDOS curves for the 10 fs cell of *a*-Si calculated using Fast, curve (**a**), and DMol3, curve (**b**). HOMOs, LUMOs and gaps are indicated.

Using the two cells constructed for amorphous silicon, the 2.44 fs and the 10 fs, we calculated the eDOS curves, the HOMOs (highest occupied molecular orbitals) and LUMOs (lowest unoccupied molecular orbitals) with both Fast and DMol3. The eDOS are shown in Figure 33 and Figure 34. For the eDOS curves obtained with Fast the discrete spectra was broadened with 0.02 eV half-width Gaussians, and for those obtained with DMol3 the broadening used was 0.05 eV.

In a first simple approach the gaps were obtained as the difference LUMO-HOMO with no attempt made to sort out the states within or near the gap due to dangling bonds and/or floating bonds. The 2.44 fs cell displayed a smaller gap than the 10 fs cell, consistent with an inhibition of the gap size with increasing defect density. This is to be expected since the larger the defect density the more ‘gap states’ which in turn reduces the overall LUMO-HOMO splitting. The numbers were potential dependent: 0.414 eV *vs.* 0.744 eV using Fast and 0.173 eV *vs.* 0.385 eV using DMol3 and although the interpretation of the electron gap as the difference of LUMOs and HOMOs may give rise to some conceptual questions, the behavior was consistent with the appearance of a narrower gap in the sample with the higher defect density.

**Figure 35:**
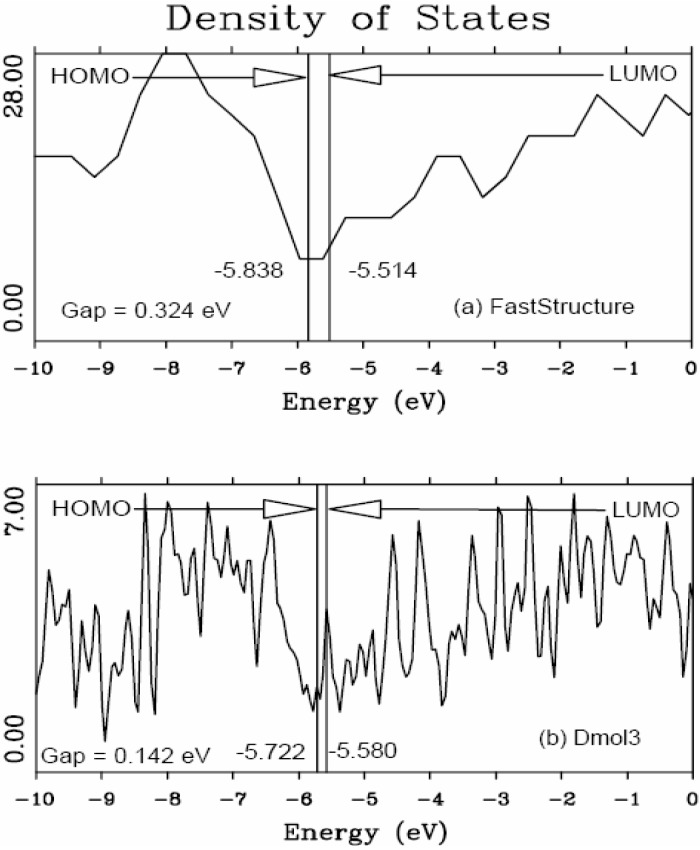
eDOS curves for the 2.44/0.46 fs cell of *a*-SiH calculated using Fast, curve (**a**), and DMol3, curve (**b**). HOMOs, LUMOs and gaps are indicated.

**Figure 36:**
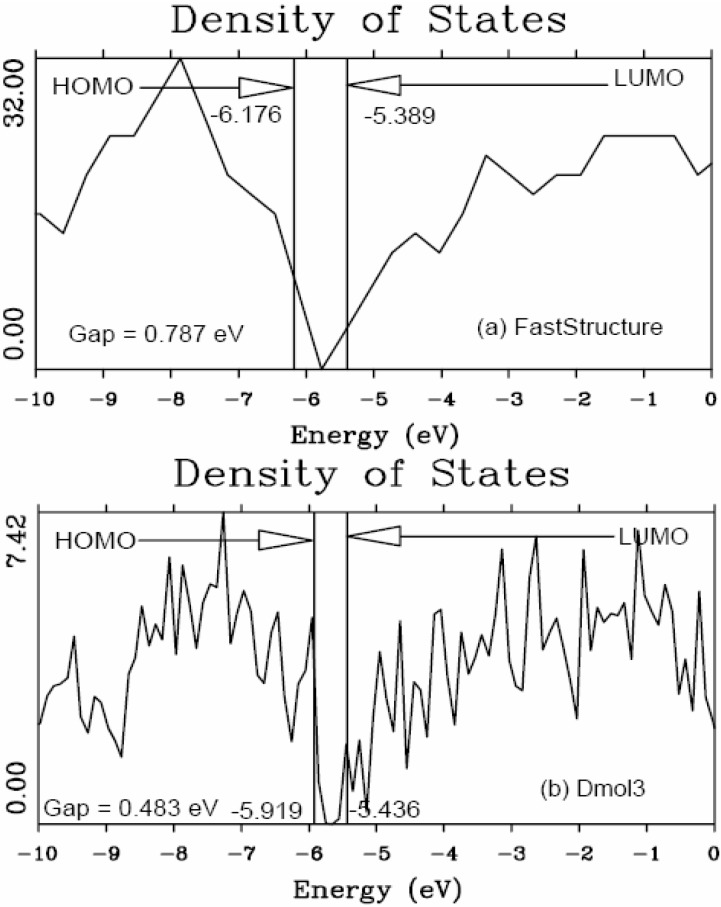
eDOS curves for the 10/2 fs cell of *a-*SiH calculated using Fast, curve (**a**), and DMol3, curve (**b**). HOMOs, LUMOs and gaps are indicated.

The two *a*-SiH cells gave rise to the eDOS shown in Figure 35 (2.44/0.46 fs cell) and Figure 36 (10/2 fs cell). Again, results are shown for the overlapped atom potential of Fast and the fully self-consistent potential of DMol3. Hydrogenation reduces the size of the gap for the 2.44/0.46 cell from 0.41 eV to 0.32 eV (overlapped atom potential) and from 0.17 eV to 0.14 eV for the self-consistent potential. For the 10/2 fs cell the gap goes from 0.74 eV to 0.79 eV with Fast and from 0.39 eV to 0.48 eV with DMol3.

**Figure 37:**
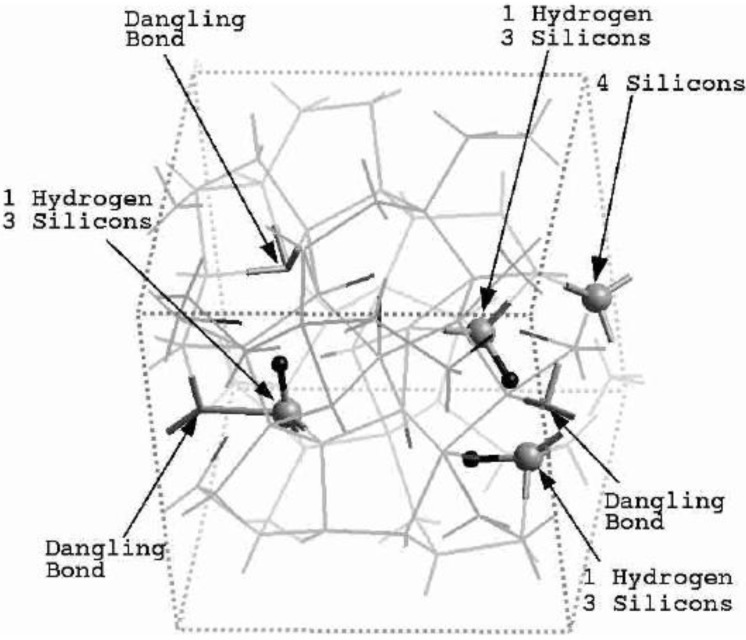
Hydrogen addition to the 2.44/0.46 cell passivates 3 dangling bonds and 2 other are passivated by silicons but 3 new ones appear; 11 new floating bonds are formed.

**Figure 38:**
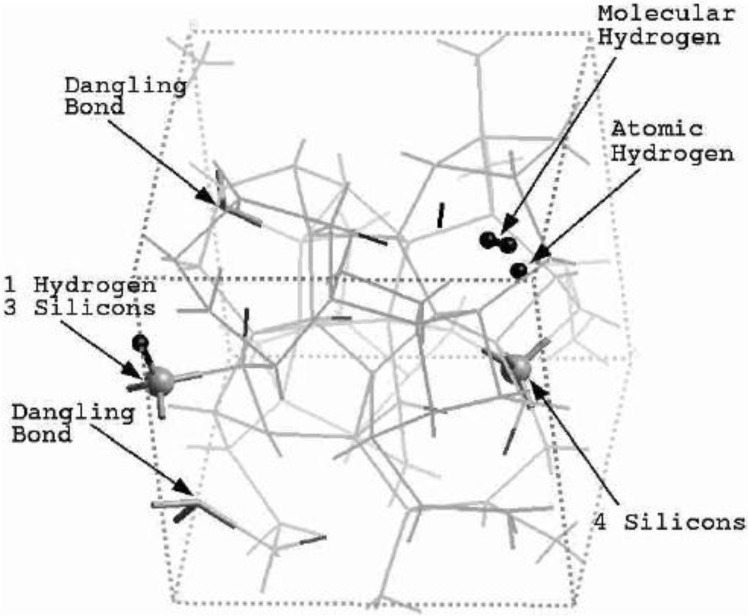
Hydrogen addition to the 10/2 fs cell passivates all dangling bonds (2) and 2 new ones appear plus 3 floating bonds. The molecular and atomic hydrogens are indicated.

The 2.44/0.46 cell has 3 dangling bonds (dbs) and 11 floating bonds (fbs) (6 hydrogen related and 5 silicon related) indicating the important role of the fbs in reducing the gap, Figure 37. The 10/2 cell has 2 dbs and 3 fbs, Figure 38, and the gap increases due to hydrogenation. An increase of the gap is also observed experimentally. Here the geometric approach to the bond length mentioned in Section 3 was used.

To conclude, the eDOS, and consequently the energy gap, showed the expected behavior with respect to the influence of defects. Upon hydrogenation of a low defect density sample, the gap was found to increase, as experimentally determined. For the high defect density on the other hand, the gap decreased; we believe due to the high number of existing fbs. In the next section (5.2.1) we present a different way to calculate the energy gap of amorphous materials after the development of Tauc several decades ago; this approach is closer to what experimentalists do when determining the optical gap.

### 5.2. Optical Gaps of Amorphous Materials *a la* Tauc. A Case Study: Amorphous Silicon-Nitrogen Alloys [1,52,53,54,55]

When dealing with amorphous silicon-nitrogen alloys we focused on the description of the atomic topology emphasizing the agreement between our simulations and the experimental RDFs reported in the literature. Even geometric aspects that we calculated, like the coordination numbers for the alloys, were in agreement with experiment. Now we present our approach to obtain values for the optical gaps of amorphous materials, based on a development by Tauc that experimentalists use commonly. Our approach was reported several years ago in References 52–55 and the gaps of our amorphous samples calculated in this manner agree well with experiment.

Experimental studies have shown that the optical gaps of amorphous silicon-nitrogen alloys depend strongly on the nitrogen content *x* for 0 ≤ *x* ≤ 1.33. It has now been established that the optical gaps of hydrogenated *a*-SiN*_x_* increase as *x* increases, slowly at first, and then close to the stoichiometric compound, *x* = 4/3 = 1.33, it attains its largest value. There are also some experimental results for the optical gap of nonhydrogenated *a*-SiN*_x_* reported by Sasaki *et al.* and Davis *et al.* [55] and the conclusions are similar.

#### 5.2.1. Optical Gaps of Amorphous Materials a la Tauc

The optical absorption coefficient *α*, the fraction of energy lost by the wave on passing through a unit thickness of the material, for interband transitions is given by:

*α* = (4*π/n_0_c*) (2*πe*^2^*ħ^3^*Ω/*m^2^*) ∫[*N(E)N(E + ħω)*|*D*|^2^*dE*]/*ħω*

as described by Mott and Davis [116]. In this equation *D* is the matrix element for transitions between states in different bands, ∂/∂*x*, which will be taken to be the same as that for transitions between extended states in the same band, (without the factor *m*/m*); *i.e.*,

*D* = *π (a/*Ω)^1/2^

where *a* is the average lattice spacing and Ω is the volume of the specimen.

One characteristic feature of optical absorption in *amorphous* semiconductors is that certain selection rules, which exist for optically induced transitions in *crystalline* materials (particularly the conservation of *k*), are relaxed.

In Mott and Davis, for interband absorption in amorphous semiconductors, the following assumptions are commonly made:

(a) The matrix elements for the electronic transitions are constant over the range of photon energies of interest and given by *D* = *π* *(a/*Ω)^½^.
(b) The *k*-conservation selection rule is relaxed. As in Mott and Davis we take the matrix element to be the same whether or not either the initial or final states, or both, are localized.

Under these conditions the optical absorption coefficient *α*, for interband transitions, is given by

*α* = (8*π*^4^*e*^2^*ħ^2^a*/*n_0_cm^2^ω*) ∫*N_V_(E)N_C_(E + ħω)dE*,

where the integration is over all pairs of states in the valence and conduction bands separated by an energy *ħω*. The refractive index *n_0_* is assumed to be independent of energy.

For amorphous tetravalent semiconductors this leads to the well-known relationship

(*α*(*ω*) *ħω*)^1/2^ = const (*ħω − E_0_*)

obtained by Tauc [117] under the assumption of parabolic bands. If the bands are not parabolic the dependence may have to be written more generally as (*α*(*ω*) *ħω*)*^p/q^* = const (*ħω − E_0_*). The parabolic bands formula has been used extensively by experimentalists to obtain the optical gap *E*_0_ by fitting a straight line to the low-energy end of the data, plotted as (*α*(*ω*) *ħω*)^1/2^ *vs.* (*ħω − E_0_*), and looking at the intersection of this line with the horizontal axis (*ħω − E_0_*).

Since *α* is proportional to the fraction of energy lost by the wave on passing through the material, this implies that it is proportional to the number of electronic transitions that go from the valence to the conduction band, *N_t_* (*ħω*). Therefore plotting (*N_t_* (*ħω*)*ħω*)^1/2^, instead of (*α*(*ω*) *ħω*)^1/2^, should give the same type of behavior as experimentally observed although with a different slope; however, the intersection with the horizontal *ħω* axis, the optical gap *E*_0_, remains unaltered.

#### 5.2.2. A Case Study: Amorphous Silicon-Nitrogen Alloys

**Figure 39:**
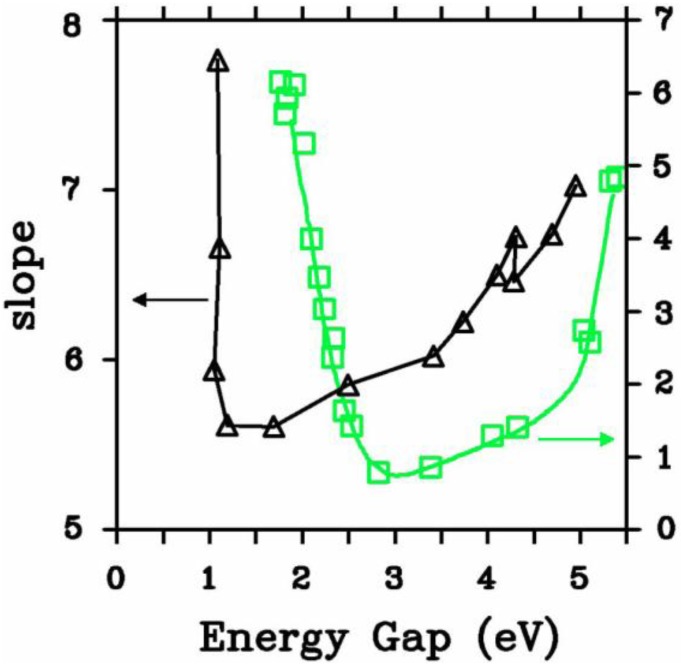
Behavior of the Tauc *slope* as a function of the gap energy. The right vertical axis, square symbols, corresponds to the experimental results due to Hasegawa *et al.* for *a*-(SiN*_x_*)H [57]. The left vertical axis, triangle symbols, corresponds to our results for *a*-SiN*_x_* using the Tauc-like approach.

**Figure 40:**
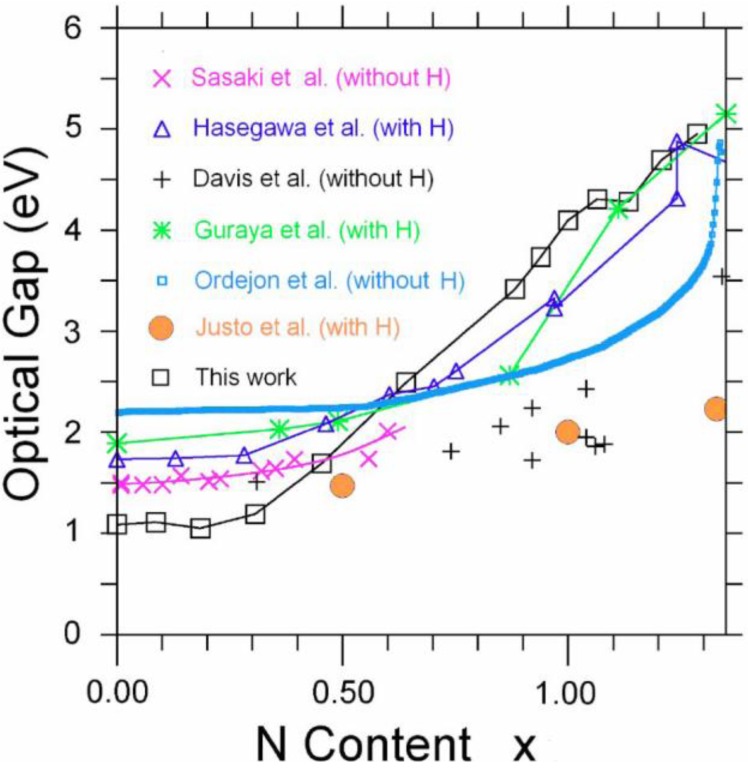
Experimental and calculated optical gaps for amorphous alloys of silicon-nitrogen for various contents *x* (see text). Lines are drawn as guides to the eye (See References [52] and [55]).

This is precisely what we do for silicon-nitrogen amorphous alloys. To find the optical gaps we look at the intersections of the linear fits to the low-energy end of the absorption curves, the (*N_t_* (*ħω*)*ħω*)^1/2^ data. The linear fits are carried out as follows: we look at the low-energy region of the absorption spectrum, 0 ≤ *ħω* ≤10 eV, and fit it with a straight line, choosing the line that gives the best linear fit for the largest number of points. The errors estimated for the slopes of these linear fits are smaller for the purer samples and increase due to the presence of states within the gap for high nitrogen contents. For example, for pure silicon the slope is 7.496 ± 0.25 and for the nearly stoichiometric sample 7.102 ± 1.11. For more detailed information we refer the reader to References 52 and 55 where results are reported that lead to the conclusion that the combination that best describes the experimental results is using a *minimal* basis set with a cutoff radius of 3 Å for the computational simulations. Based on this approach we calculated the absorption slopes and the optical gaps and these are shown in Figure 39 and Figure 40, respectively.

Figure 39 shows the behavior of the Tauc *slope* as a function of the gap energy. The right vertical axis, square symbols, is related to the experimental results due to Hasegawa *et al.* for glow discharged *a*-(SiN*_x_*)H [57]. The left vertical axis, triangle symbols, refers to our results for *a*-SiN*_x_* using the Tauc-like approach. It can be observed that the slope of our Tauc fit diminishes first and increases afterwards, as a function of the energy gap, as experimentally reported by Hasegawa *et al.* In order to compare the experiment and our simulation we have plotted the average slope (which is the average of the values of each of our two runs), as a function of the energy gap, using a vertical coordinate given by (*N_t_* (*ħω*)*ħω*)^1/2^/*ħω*. The experimental results that Hasegawa and co-workers report are given in units of 10^5^ eV^−1^ cm^−1^, so the most we can hope for is the qualitative comparison of both results given in Figure 39. The similarity is quite impressive, even though the experimental results are for hydrogenated *a*-SiN*_x_* samples, indicating that our approach seems to be along the correct lines.

In Figure 40 we plot the optical gaps of several experimental and theoretical results reported in the literature. The experimental hydrogenated results are due to Hasegawa *et al.* and Guraya *et al.* The experimental nonhydrogenated gaps are due to Sasaki *et al.* and Davis *et al.* The optical gaps, calculated using our approach, are of the correct order of magnitude and behave very similar to experiment. The simulations of Ordejón *et al.* for samples without hydrogen, an atomistic calculation with adjusted parameters, are also included. The optical gaps, calculated using our approach, are of the correct order of magnitude and behave very similar to experiment. Our gaps are below all results for *x* < 0.5 and above all results for *x* > 0.6. For *x* ≈ 1.3 experiments and simulation become indistinguishable; the behavior clearly changes for *x* ≈ 1.1, the percolation threshold of Si–Si bonds [52,55].

To conclude, our Tauc-like approach gives good results when calculating the slope of the optical absorption curve, Figure 39. It also gives good results when finding the optical gaps, Figure 40. We believe that the good agreement is directly related to the realistic representation of amorphous materials by the random networks generated according to our computational protocol.

### 5.3. Vibrational Properties in the Harmonic Approximation [118]

The study of the frequency spectrum of a condensed system, and its corresponding vDOS, is an important and direct manner to analyze whether a given structure is in fact a minimum energy structure, since it is known that the presence of ‘negative’ frequencies signals instabilities in the system considered. In fact, what is negative is the square of the frequency, which leads to an imaginary value for ω. Clearly, an amorphous material is not the minimum energy structure of the solid since their crystalline counterparts have the lowest energy arrangement. Therefore, at best we hope to find our amorphous sample in a local energy minimum and some negative frequencies are expected. Also, when calculating the frequency spectrum care has to be exercised to consider very small displacements around the equilibrium position of the atoms, so that the harmonic approximation is applicable; otherwise anharmonic considerations have to be included.

When experimental results exist then it becomes an important benchmarking exercise to simulate the corresponding amorphous material and calculate its vDOS. If the RDFs, the electronic structure (eDOS), the optical gap and the vDOS agree with experiment then we can say, with some certainty that the computationally generated atomic structure is close to the experimental one. For pure silicon we generated a 216-atom amorphous supercell using the *undermelt-quench* approach and calculated its vDOS. The results that our group has obtained for the vibrational spectrum of pure amorphous silicon show the best agreement with experiment found so far in the literature [118]. Work is in progress to calculate the vDOS, and any other property that may be relevant, for all the networks that we have generated in order to see how good the topologies of our amorphous samples are. For a more detailed discussion of these results we refer the reader to Reference 118.

**Figure 41:**
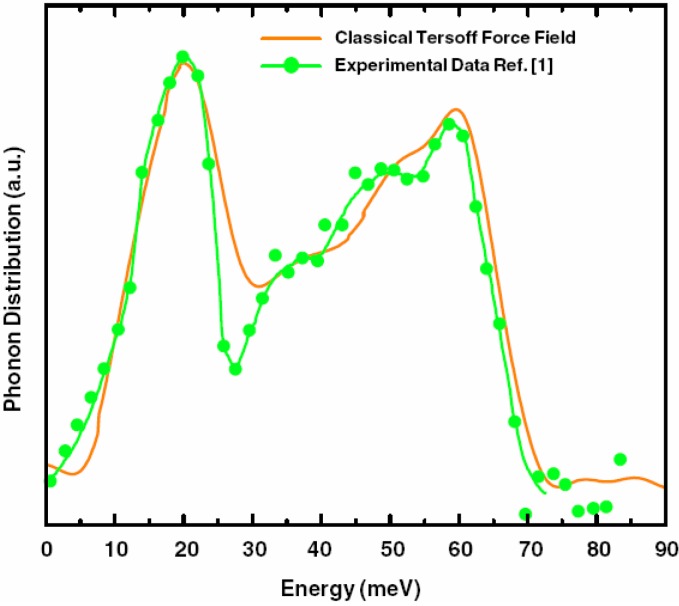
Classical vDOS, based on the Tersoff potential [119], for our 216-atom *a*-Si network (orange curve), compared to the experimental results of Kamitakahara *et al.* (green curve and dots) [120,121].

**Figure 42:**
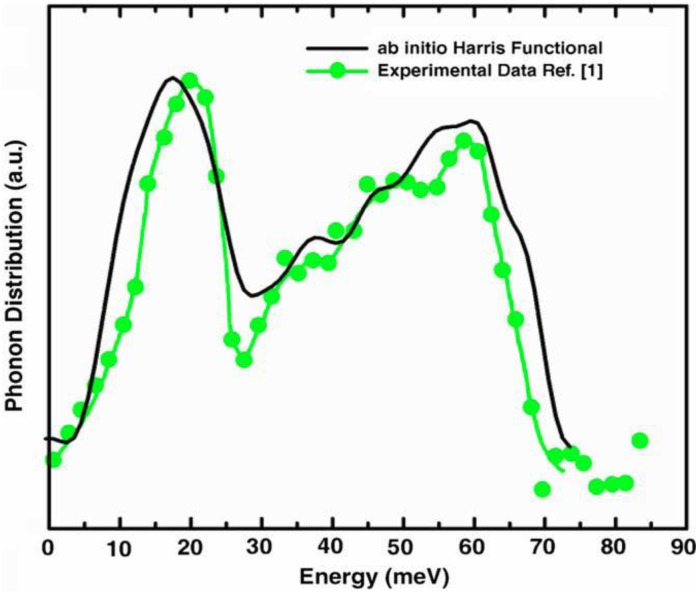
Quantum vDOS for our 216-atom *a*-Si network (black curve), compared to the experimental results of Kamitakahara *et al.* (green curve and dots) [120,121].

**Figure 43:**
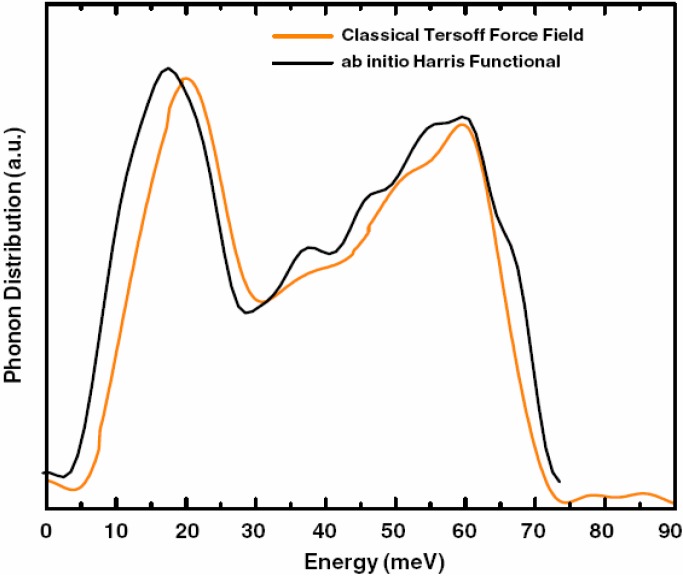
Comparison between the quantum (Harris, black curve) and the classical (Tersoff, orange curve) vDOS for the 216-atom *ab initio* generated *a*-Si network [118].

Our results, the comparison with each other and with experiment, are shown in Figure 41, Figure 42 and Figure 43. Figure 41 is the comparison between our calculation, based on the classical Tersoff potential [119] and done with the help of the OXON package [122], and experiment [120,121]. Figure 42 is the comparison of experiment with our *ab initio* result. Figure 43 is the comparison of our two theoretical results, one obtained with the OXON package (Tersoff potential) and the other with the Materials Studio Modeling package (*ab initio* approach). It is clear then that our results, both classical and quantum, agree quite well with experiment; they are the closest to the experimental data found in the literature.

To conclude:

- Our frequency calculations are based on the 216-atom random structure that we *ab initio* generated using the *undermelt-quench* approach + the code Fast.
- The relative heights of the two prominent peaks of the vDOS from both experiment and simulations essentially coincide and so do the overall shapes.
- The positions of the two prominent peaks are essentially the same for the simulations and experiment. The first peak of the Harris calculation is somewhat displaced towards lower energies.
- In the region 30–50 meV the Harris simulation agrees better with experiment than Tersoff’s.

The agreement of our classical and quantum results with experiment is better than any reported in the literature, classical or *ab initio*. This agreement indicates that our 216-atom amorphous structure adequately describes the material used in References 120 and 121; it also indicates that the agreement we have found using our amorphous supercell for both methods of calculation manifests the ‘correctness’ of the amorphous structure of our supercell. For the crystalline materials it is common to refer to the four major peaks of the vDOS as the transverse acoustical (TA), longitudinal acoustical (LA), longitudinal optical (LO) and transverse optical (TO) peaks. Clearly, for an amorphous material this classification is not as evident as it is for the crystalline counterpart. Nevertheless it is argued that these four major peaks in the vDOS of the crystal also appear in the vDOS for *a*-Si and that there is, overall, a substantial similarity between the two distributions. To us there are important differences between the vDOS of the two phases, like the position of the peaks and their relative intensities, and also the presence of an abundant number of low frequency modes that do not exist in the crystalline counterpart, to mention but a few.

## 6. Conclusions

Simulations generate well defined amorphous atomic networks which may or may not be close to the real structure of the amorphous material. Experiments start from real structures, but with no knowledge of the detailed atomic arrangement. The RDF is one bridge between the two. Other bridges are the physical properties that, when calculated based on the detailed atomic structures of the simulated materials, may shed light on the adequacy of the generated atomic topology to describe the real amorphous material.

The fact that our calculations agree with experiment, as has been clearly shown throughout this work, makes us reasonably optimistic that our protocol, the *undermelt-quench* approach (and its variants) + Fast, may be adequate for the generation of “true” atomic topologies of amorphous material. Also, the Tauc-like approximation that we have devised to obtain the optical gaps of amorphous semiconductors seems to be adequate to describe these properties and perhaps can be generalized to describe other materials with non-parabolic bands. Multicomponent amorphous alloys of semiconductors and metals, and BMGs are amenable to our approaches; some of them are presently being studied and will be reported in the future.
